# Multi‐Target Wireless Localization of Miniaturized Magneto–Oscillatory Trackers in Soft Biological Tissues

**DOI:** 10.1002/advs.78241

**Published:** 2026-11-09

**Authors:** Liyuan Tan, Jyothi Kumari Mariyanna, Meng Zhang, Alexander Kutscher, Ann‐Sophia Müller, Felix Fischer, Tian Qiu

**Affiliations:** ^1^ Division of Smart Technologies for Tumor Therapy German Cancer Research Center (DKFZ) Site Dresden Dresden Germany; ^2^ Institute of Semiconductors and Microsystems, Faculty of Electrical and Computer Engineering Dresden University of Technology (TUD) Dresden Germany; ^3^ Faculty of Medicine Carl Gustav Carus Dresden University of Technology (TUD) Dresden Germany; ^4^ Faculty of Computer Science Dresden University of Technology (TUD) Dresden Germany

**Keywords:** computer‐assisted intervention, magnetic localization, miniaturized wireless trackers, tissue viscoelasticity

## Abstract

Accurate lesion localization is essential for precision medicine applications such as radiation therapy and minimally invasive surgery. However, preoperative imaging often fails to represent intraoperative anatomy because of surgical repositioning and physiological motion, highlighting the need for a wireless, radiation‐free tracking approach with real‐time positional feedback. Here, we present miniaturized wireless trackers based on a small‐scale magneto–oscillatory localization (SMOL) method for multi‐target localization in soft tissues. Distinct resonance frequencies are achieved using micro‐resonators with different cantilevers and numbers of attached micro‐magnets, enabling simultaneous identification and tracking. An optimized excitation strategy using three electromagnetic coils and a multi‐step search scheme allows localization without prior target position or orientation information. The system achieves sub‐millimeter precision and accuracy below 2 mm at distances up to 115 mm. Continuous tracking of three independent trackers was demonstrated in soft tissue phantoms and *ex vivo* porcine liver tissue. In addition, oscillation damping behavior indicates potential for rheological sensing of soft tissues. These wireless miniaturized trackers show strong potential for real‐time localization in computer‐assisted interventions.

## Graphical abstract

Miniaturized, frequency‐encoded magneto–oscillatory trackers enable wireless localization of multiple targets without prior knowledge of their positions or orientations. The method achieves accurate localization over clinically relevant distances and measures local viscoelasticity via the oscillatory responses. It provides a versatile platform for multi‐target wireless localization and mechanical sensing in deep biological tissues.

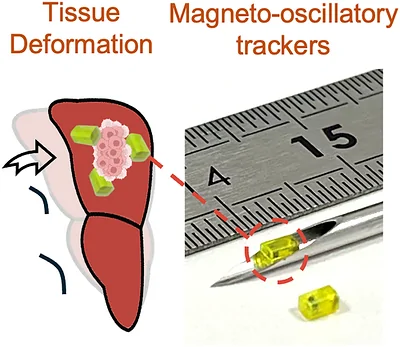

## 1 Introduction

In vivo localization and tracking of tumor or lesion site is a central requirement for precision radiation therapy [1, 2, 3], minimally invasive surgery [4, 5, 6], and targeted drug delivery [6, 7, 8]. Accurate knowledge of their position over time enables clinicians to align preoperative imaging with intraoperative anatomy, maintain safe tool‐to‐target distances, and compensate for motion induced by respiration, cardiac activity, or surgical manipulation [9, 10]. However, pathological targets are often small, deformable, and embedded in moving organs, so their apparent position in the imaging frame can deviate from the preplanned trajectory. Organs such as the liver, lung, pancreas, and adrenal gland can undergo substantial posture‐dependent deformation and gravity‐induced displacement, leading to large localization errors relative to preoperative imaging; this issue is clinically relevant in many indications, such as Hepatocellular Carcinoma, Lung Adenocarcinoma, Pancreatic Carcinoma, and Pheochromocytoma [11, 12, 13, 14]. Figure 1A illustrates an example of organ position change and deformation of liver due to the change of surgical position, from the supine position to the lateral decubitus position, with Figure 1B demonstrating the use of trackers for organ deformation with a magnified view. While other positions, like the latter position, are normally seen in surgical procedures, the supine one is also typically used in most medical imaging methods [15, 16], which introduces a gap between static preoperative information and dynamic intraoperative reality.

**FIGURE 1:**
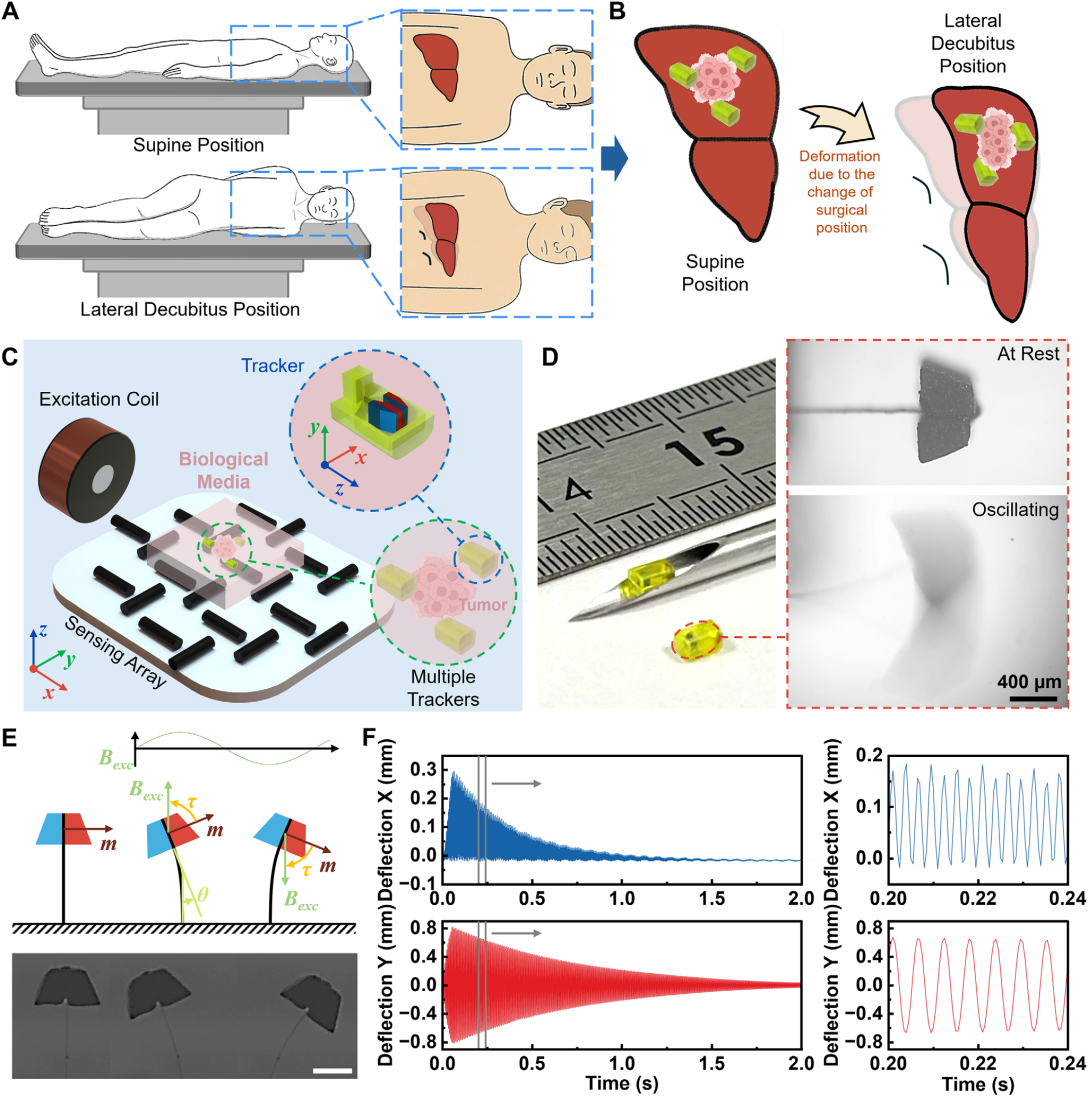
Overview and schematic of using the miniaturized wireless trackers for multi‐target localizations in biomedical applications. (A) Organ deformations due to the change of surgical positions. (B) Magnified view of the deformed organ with multiple trackers to track the lesion position. (C) Overview of the tracking system with electromagnetic coils for tracker excitation and a sensing array for receiving signals. (D) A picture of the miniaturized wireless tracker using the small‐scale magneto–oscillatory localization (SMOL) method. The inset shows an example of the oscillator embedded in the protective housing at rest and at the oscillating state. (E) Excitation principle of the magnetic oscillator inside the tracker with a magnetic moment perpendicular to the plane of the cantilever. Scale bar: 500 μm. (F) Optical tracking of the centroid of a typical oscillator. The plots on the right represent a zoomed‐in view of the gray regions on the left.

In addition to lesion tracking, emerging minimally invasive tools, such as microrobots [17, 18, 19, 20], flexible catheters [21, 22, 23], and other targeted drug delivery devices [24, 25, 26], also critically depend on accurate in vivo localization. These small‐scale systems often operate within confined, deformable, and dynamic anatomical environments, ranging from soft tissues to luminal organs, where direct visualization is limited or impractical [27]. Tracking of their position and orientation is essential for guiding interventions, avoiding healthy tissue, and enabling feedback control during navigation [28, 29]. As these technologies move toward broader clinical translation, the demand for reliable, high‐resolution localization methods becomes essential to ensure safety and effectiveness in both diagnostic and therapeutic procedures.

To meet these localization demands during clinical procedures, intraoperative imaging methods are often employed to provide surgeons with timely anatomical and pathological information. However, each modality has practical considerations that may limit its use for continuous or repeated intraoperative localization. For example, intraoperative Computed Tomography (CT) offers high spatial resolution and geometrically accurate volumes, but its acquisition typically interrupts the surgical workflow and introduces additional ionizing radiation [30, 31, 32]. Image quality can also be degraded by metallic implants and by the constrained position of the C‐arm in the operating room [30, 32]. Although ultrasound (US) imaging is radiation‐free, portable, and capable of real‐time repetition at the bedside, its diagnostic and navigational value is highly operator‐dependent since the image quality varies with probe orientation, acoustic window, and local tissue conditions, and is often compromised near gas, bone, or in narrow surgical corridors. Moreover, conventional US imaging is predominantly slice‐based, while 3D US requires additional acquisition and reconstruction strategies and remains dependent on acoustic access and image quality. Magnetic Resonance Imaging (MRI) is also a common method for clinical localization. However, it is typically used for preoperative location determination. Other modalities, such as positron emission tomography (PET) and X‐ray fluoroscopy, also involve ionizing radiation, for which radiation exposure is an important consideration when repeated or prolonged imaging is required. These imaging modalities provide indispensable anatomical and/or functional information, but each has different trade‐offs in terms of radiation exposure, temporal resolution, accessibility, and compatibility with continuous intraoperative localization. Taken together, these considerations motivate the development of complementary localization approaches capable of long‐term and repeated 3D tracking of tumor or lesion positions during surgery. Such capabilities could further improve surgical precision, minimize damage to surrounding healthy tissues, and ultimately improve patient outcomes.

To realize 3D localization or tracking of the tumor or lesion position in the human body with minimized radiation, localization methods utilizing magnetic fields are promising, with high temporal and spatial resolutions in deep tissues, and are safe inside the body. Recent advances in magnetic localization have enabled the tracking of untethered medical devices using embedded magnets and external sensors, offering radiation‐free and real‐time solutions in vivo. For example, gradient‐based methods have been used to achieve sub‐millimeter positional accuracy within surgical‐scale workspaces using compact magnetic chips that are suitable for intraoperative navigation [33]. Marker‐based systems such as MOLLI utilize ferromagnetic seeds and handheld probes to assist in the precise localization of nonpalpable tumors during surgery [34]. Several 5∼6‐degree‐of‐freedom (DoF) systems based on static dipole models and Hall sensor arrays have enabled high‐speed tracking of capsule endoscopes and microrobots, but they typically require dense sensing arrays and have limited effective range [35, 36, 37]. More recent works have explored adaptive localization–actuation coupling for confined tubular environments and wireless nuclear magnetic resonance‐based tracking for sub‐millimeter‐scale implants with nT sensitivity [38, 39]. A distinct approach employing rotating magnetic dipoles generated by miniature synchronous motors with thermal locking mechanisms, achieving 3D pose estimation via a single triaxial Hall sensor and supporting integrated manipulation and tracking [40], has been proposed. However, many of these systems still face challenges in achieving full 6‐DoF, maintaining compact sensor design, and supporting multi‐target operation. Resonant magneto‐mechanical approaches have been proposed to enable high‐sensitivity 6‐DoF tracking using symmetry‐breaking oscillatory magnetic responses [41]. In parallel, the small‐scale magneto–oscillatory localization (SMOL) method follows a similar resonance‐based strategy but preserves an effective magnetic moment for potential actuation. The SMOL device has demonstrated a localization resolution of up to sub‐100 μm, a working distance of up to 12 cm, and a refresh rate of 5 Hz [42]. However, the previous design of the SMOL device has a footprint of 4 mm × 3 mm × 2.4 mm (∼14 ${\mathrm{mm}}^{3}$), and therefore it is desired to further miniaturize the device for broader biomedical applications, especially for minimally invasive procedures. Moreover, it is also important for such systems to support multi‐target localization, as tumors or lesions often span a relatively large 3D volume that requires localization at multiple spatial points for more accurate mapping.

In this paper, miniaturized wireless trackers capable of measuring localized rheological properties at the corresponding location are fabricated for localization and tracking in biological tissues at multiple targets, based on the SMOL methods using an embedded micro–magneto–mechanical oscillator, as shown in Figure 1C. A 20‐sensor sensing array is designed to improve the localization space and accuracy, while the excitation of the trackers is realized via electromagnetic coils. The miniaturized trackers with a size of 1.5 mm ×1.5 mm ×2.2 mm are fabricated and can be inserted into biological tissues using a 12‐gauge (12G) needle, as shown in Figure 1D, with the micro–oscillators enclosed by a protective housing. The oscillator with attached micro‐magnets has a magnetic moment perpendicular to the cantilever and, therefore, can be excited by an alternating magnetic field along the cantilever (Figure 1E). The oscillator will undergo free oscillation until it is fully at rest due to damping from the environment after a short period of excitation. Figure 1F presents the deflection distance of a sample oscillator from excitation to free oscillation. Individual trackers with different cantilever thicknesses and types are characterized, and localizations with a single tracker are performed to verify their performance with high precision and accuracy up to 115 mm. Multi‐target localizations are first characterized with three coaxially excited trackers. Then, continuous tracking of three off‐axis trackers is demonstrated with a custom‐built proof‐of‐concept 3D excitation coil platform, and excitation is achieved without initial position and pose information. Moreover, the possibility of using the trackers as a miniaturized rheometer for localized rheological measurement is demonstrated since the resonant frequency and damping rate of a tracker are highly dependent on the environment surrounding the tracker. Last, multi‐target localization in an *ex vivo* porcine liver is realized with a strong increase in the damping rate considered. Building upon the successful demonstration of miniaturized wireless trackers for multi‐target localization in biological tissues, this work further establishes a platform that enables deep‐tissue localization of small‐scale robots or medical instruments, thereby opening new opportunities for advanced biomedical interventions and future clinical translation.

## 2 Results and Discussion

### 2.1 Fabrication and Characterization of the Miniaturized Wireless Trackers

A tracker mainly consists of a micro–magneto–mechanical oscillator and a polymeric protective housing. All components, including the magnets, the cantilever, and the housing, were assembled manually following a step‐by‐step assembly process, as shown in Figure 2A. The tracker has an oscillator with an attached mass that contains two types of trapezoidal micro‐magnets. The two types of micro‐magnets have opposite magnetic moment directions relative to the geometry, e.g., one towards the perpendicular side edge, while the other towards the slanted side edge with the north and south poles shaded in red and blue, respectively, as in Figure 2A(i). To exploit the frequency response of the tracker, two types of trackers are prepared based on the number of micro‐magnets, i.e., with two or four micro‐magnets (labeled as 2M and 4M, respectively). To obtain oscillators with four micro‐magnets, two different types of the aforementioned trapezoidal magnets are first forced to form a hexagonal micro‐magnet with identical pole directions, as shown in Figure 2A(i). This arrangement has multiple advantages: first, it maximizes the magnet volume for a stronger signal generation; second, it provides an additional approach to adjust the oscillator properties, i.e., resonant frequency and damping rate; third, the lower magnets have the perpendicular sides parallel to the housing boundary and therefore will not affect the achievable oscillation amplitude comparing with the two‐magnet case; forth, the slanted sides of the lower magnets provide openings for the bending motion of the cantilever during the oscillation and will not affect the overall length of the cantilever. The following assembly steps are shown in Figure 2A(ii)–(v), with the detailed process provided in the Experimental section.

**FIGURE 2:**
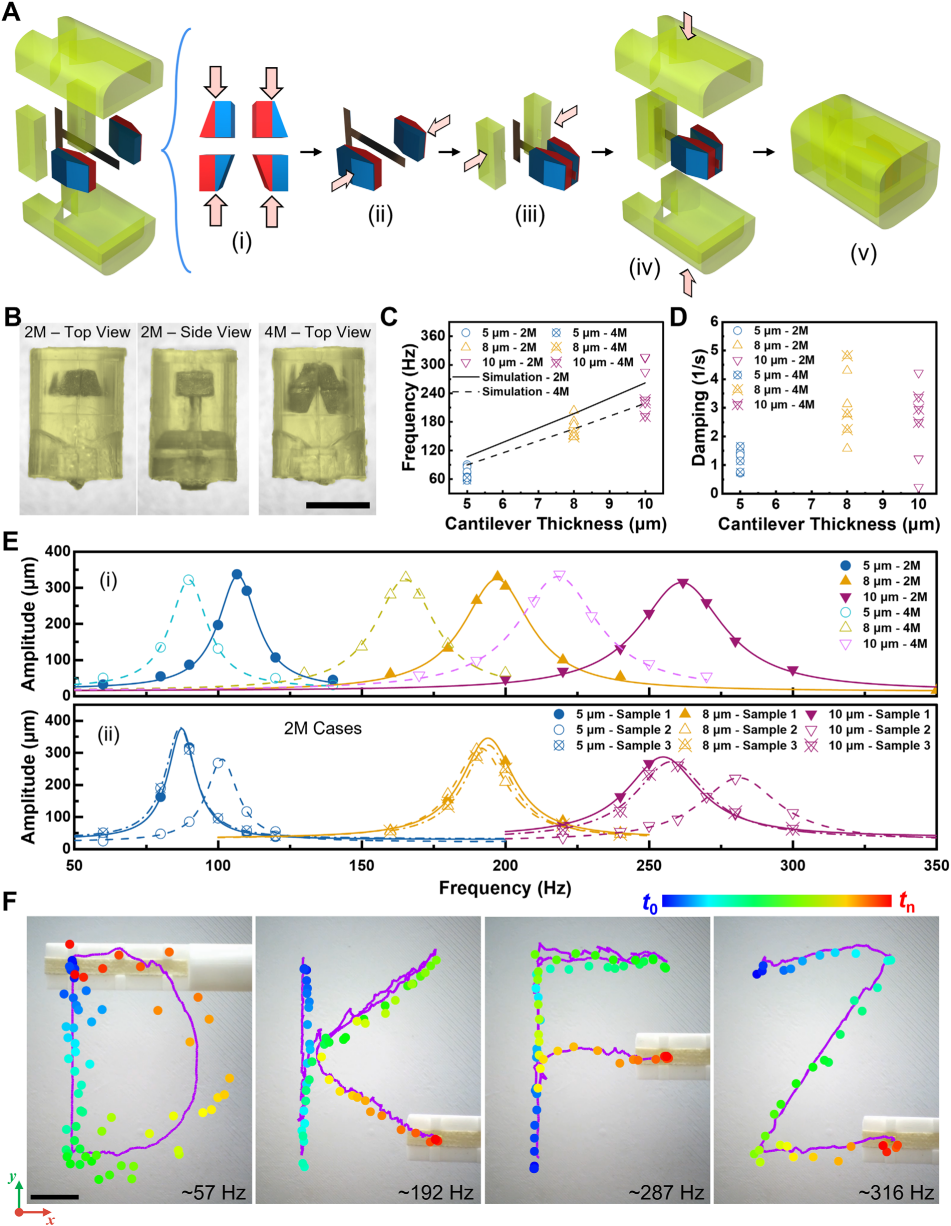
Fabrication and characterization of trackers with various designing parameters for selective excitation. (A) Fabrication process of the trackers. (B) Two tracker examples with 2 and 4 attached magnets. Scale bar: 1 mm. (C,D) Calibrated frequency and damping rate of the trackers (N=3). (E) Simulation results of the oscillating amplitude of the device with different cantilever thickness and magnet number at various excitation frequencies, giving a magnetic field to reach the maximum allowed oscillation amplitude after 10 periods of excitation (top row). Experimental results of 2M‐typed oscillators with different cantilever thicknesses at various excitation frequencies, giving a magnetic field obtained from the simulation results that achieves the maximum allowed amplitude (bottom row). Each cantilever thickness is tested with three oscillators. F) Tracking of different trackers with pre‐defined trajectories of letters of DKFZ, on the plane of z = 54 mm. Scale bar: 10 mm. See also Video S1.

The two types of fabricated trackers with two and four micro‐magnets are shown in Figure 2B with top and side views. In this study, cantilevers with three different thicknesses but identical length and width are used. Therefore, a total of six trackers with different performances are used. Finite element simulations of the oscillator's behavior are performed and compared with the measurements using a high‐speed camera. Figure 2C,D show the natural frequencies ($f_{nat}$, Hz) and damping rate (η, 1/s) of the fabricated trackers, respectively. $f_{nat}$ increases with cantilever thickness for both the 2 and 4M types, while the 4M type exhibits a lower $f_{nat}$ as the attached mass is larger. Finite element simulations of the $f_{nat}$ with the two types of oscillators with different cantilever thicknesses show good agreement with the measured results. While the $f_{nat}$ shows a more significant trend, the trend of the η is not that obvious. The low $f_{nat}$ trackers with a 5 μm in general give a low damping rate with a narrow distribution, while the higher $f_{nat}$ ones show a higher average η and a larger distribution compared with the trackers with a 5 μm thick cantilever. The dependence of the resonance frequency on the cantilever length is shown in Figure S1 through simulation, where the length plays a dominant role by governing the effective structural stiffness. However, in this work, the resonance frequencies are mainly tuned by adjusting the thickness of the cantilever.

Selectivity tests of the oscillators are investigated experimentally and by simulation as shown in Figure 2E. Based on the housing design which allows an oscillating amplitude from the centroid of the attached mass of ∼300 μm, finite element simulations are applied to all 6 designs of the oscillator to investigate the minimum field requirements to drive them to the desired amplitude (∼300 μm) with 10 cycles of excitation at resonance. The minimum fields required for oscillators with cantilever thicknesses of 5, 8, and 10 μm are 40, 120, and 200 μT, respectively, for the 2M cases, as a thinner cantilever requires less force to bend. For the 4M cases, the corresponding minimum field requirements are 25, 70, and 120 μT, as a larger attached magnet has a larger magnetic moment and thus a larger torque for bending. In general, an oscillator with a high $f_{nat}$ typically needs a stronger field to excite. However, for a certain excitation coil, it is more difficult to excite such an oscillator because of the induction at high frequencies. A much higher voltage is needed to reach the same field compared with a low‐frequency one. Therefore, using a high $f_{nat}$ oscillator can easily drive the voltage or power to the limit of the powering system. However, since the SMOL methods process signals with their derivative, a high $f_{nat}$ oscillator is, to some extent, preferred because it provides a higher signal‐to‐noise ratio. Thus, there will be a trade‐off between the sensing and the excitation distances. Figure 2E(ii) compares the simulation results with experiments for the 2M cases. While most fabricated oscillators show perfect agreement with simulations, some deviations can be seen due to manufacturing defects.

To evaluate the localization and continuous tracking performance of the fabricated trackers, multiple trackers with different $f_{nat}$ values were tested along predefined trajectories that outline the letters of “DKFZ”, as shown in Figure 2F and in Video S1. The trackers are coaxially excited. In general, all four cases demonstrate their capability of localization and continuous tracking. However, the D trajectory exhibits lower accuracy due to its low $f_{nat}$, which leads to a reduced signal‐to‐noise ratio. In contrast, the other three trajectories, achieved with higher $f_{nat}$ trackers, demonstrate improved accuracy, indicating a clear preference for higher $f_{nat}$ designs.

### 2.2 Characterization of Multi‐Target Localization with Multiple Trackers

Since the trackers include oscillators with hanging magnets, it is crucial to understand the distance limit of using multiple trackers simultaneously. If the two trackers are too close, the magnets inside the trackers will attract each other and apply external forces onto the oscillator, and the trackers' performance will be unpredictable. Therefore, a finite element simulation is performed to determine the safe distance between the trackers. A magnet set identical to the 2M case is positioned at different distances and angles to a 2M oscillator and the forces applied to the oscillator are obtained as shown in Figure S2. We find that the interaction force drops to a steady level close to zero when the centroid‐to‐centroid distance is 4 mm. Given that the oscillators can bend within the housing limits, a 5 mm gap is required. For the 4M cases, as the force increases fourfold and the force between them is inversely proportional to $r^{4}$, the required distance is ∼7 mm to maintain the same force level considered as the absence of interaction. Therefore, the trackers tested in the following sections are kept at a safe distance of at least 10 mm.

To demonstrate the feasibility of using the trackers for multi‐target localization, three trackers, with $f_{nat}$ of ∼185 Hz, ∼220 Hz, and ∼287 Hz, labeled as Tracker 1, 2, and 3, respectively, are selected and excited coaxially with one common excitation coil for characterization with an excitation field perpendicular to the magnetic moments of the trackers. To avoid signal overlap from different trackers and attenuate noise components from the target signal, a band‐pass filter with a ±20 Hz bandwidth around the target frequency is used, along with two notch filters to remove frequency components from the other two trackers. However, applying such a filter results in transient responses of the measured signal; therefore, the first 44 ms of the signal has to be removed. The loss of signal amplitude during this period will be compensated based on the calibrated damping rate η.

All experiments were performed using a 20‐sensor sensing array, as shown in Figure 3A, with the schematic of the testing platform above the sensing array shown in Figure 3B. The three trackers are aligned coaxially with a spacing of 10 mm (Figure 3C). To verify whether the relative pose of the trackers introduces any deviations, they are tested with various angles about the tracker's local z axis, with the rotation of angles defined in Figure 3D. The trackers are first tested at Z=54 mm above the sensor platform with different rotation angles, and the localization results are provided in Figure 3E,F for positions and poses, respectively. The localization is very stable with small standard deviations, and the deviation from the ground truth remains below 2 mm depending on the pose. The measured local z‐axis orientation agrees well with the ground truth rotation angle, while the measured misalignment (misalignment between the local z axis and the global x axis) remains consistent within approximately 5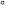 during rotation. These results demonstrate stable localization of the miniaturized trackers at representative positions and orientations. The 6‐DoF pose estimation follows the previously established SMOL localization framework [42], in which the measured multi‐sensor magnetic signals are fitted to the dipole model by jointly optimizing the three‐dimensional position (x,y,z) and orientation represented by a unit quaternion. Therefore, the experiments presented here provide representative validation of the miniaturized trackers rather than a comprehensive re‐validation of the full 6‐DoF localization framework.

**FIGURE 3:**
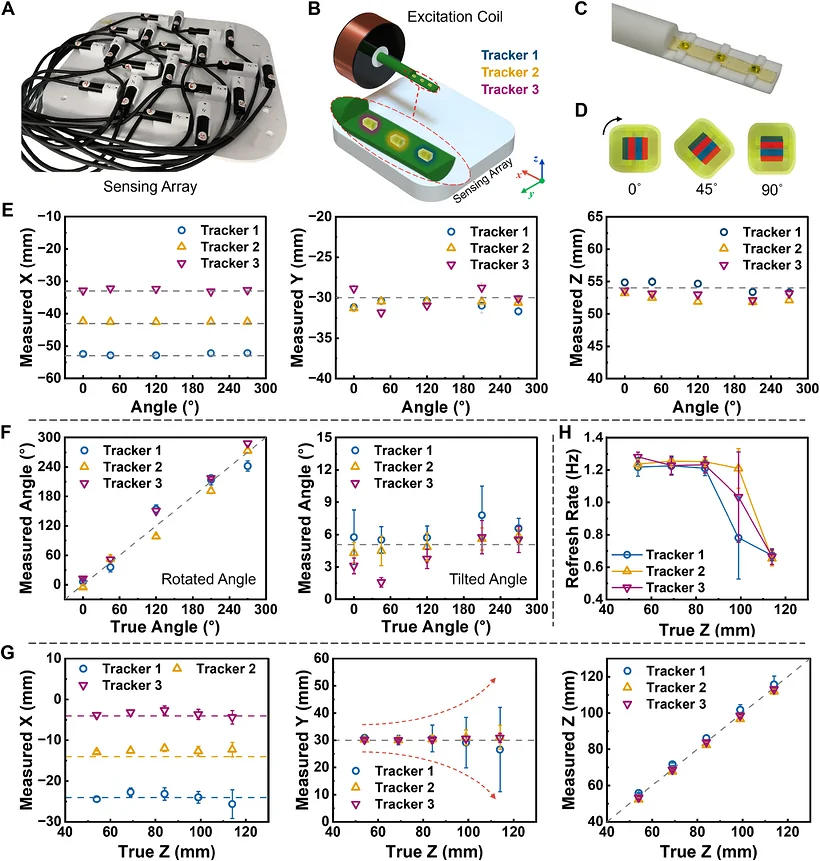
Characterizations of multiple trackers for multi‐target localization. (A) Photograph of the 20‐sensor sensing array used for magnetic signal acquisition and tracker localization. (B) Schematic of excitation with three trackers aligned to the axis of the excitation coil and parallel to the x axis of the sensing array. (C) Photograph of the coaxial testing setup for experimental characterization of the trackers. (D) Schematic of the change of relative dipole field to the sensing array by rotating the tracker about its local z axis. (E) Localization results of three trackers aligned to the x axis of the sensing array with different rotational angles described in (D) at y=−30 mm and z=54 mm. (F) Measured angles with the same poses as in (E), with the rotated angle indicating the rotation about the local z axis and the tilted angle indicating the misalignment between the setup and the global x axis. (G,H) Localization results and refresh rates of the trackers at various heights above the sensor plane. All results are presented with N=20.

Subsequently, the performance of the trackers is also tested at different heights. As shown in Figure 3G, the average localization results match the true position. However, the higher the tracker is above the sensor platform, the larger the standard deviation. This is due to the low signal‐to‐noise ratio at higher positions, and the real‐time measurements are therefore strongly affected by the random noise at the same frequency that cannot be effectively filtered. Nevertheless, the trackers with a higher $f_{nat}$ show better performance, with Tracker 2 providing accurate measurements up to Z=100 mm and Tracker 3 maintaining accuracy beyond Z>110 mm. This is expected as the SMOL method processes the gradient of the measured field; the trackers are more resistant to noise with a high $f_{nat}$. Tracker 1 only shows good performance when the height is below Z=70 mm. The errors across all tested heights remain small and are mainly reflected in the measured X and Y coordinates. However, it is possible for accurate static localization even with Tracker 1, provided that sufficient data are collected over time, since the average measured locations are accurate. The above findings are also verified by performing the localization for trackers aligned at 45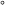 toward the center at different heights Z, as shown in Figure S3. The results agree with the above measurements, with standard deviations increasing with Z, especially for Tracker 1. The refresh rate, as presented in Figure 3H, remains relatively stable (∼1.2–1.3 Hz) at lower Z, but drops noticeably beyond ∼90–100 mm, indicating degraded tracking performance at larger distances. While Trackers 2 and 3 maintain high localization accuracy, the refresh rate decreases, likely due to the reduced signal amplitude, which weakens the effective excitation response and degrades the stability of the optimization.

The above characterizations were conducted under ideally aligned excitation conditions to maximize the perpendicular component of the excitation field to the tracker's magnetic moment. In practical scenarios, excitation misalignment may still occur, leading to reduced oscillation amplitude. This effect can be compensated by increasing the excitation field strength or the number of excitation cycles to ensure sufficient excitation. Detailed characterization is provided in the Supporting Information.

### 2.3 Multi‐Target Localization without Initial Information

The localization of multiple targets has been successfully demonstrated, provided with appropriate excitation, in the last section. However, the initial positions and poses of the trackers injected into an organ are unknown, especially when there are organ deformations. Here, a multi‐step localization strategy is proposed, consisting of three steps. First, space scanning is performed using fields along multiple directions uniformly distributed over a semi‐sphere. The directions are selected to cover the semi‐sphere based on the Fibonacci spherical sampling method, with a prescribed angular separation, as illustrated in Figure S6. In this step, the orientations of the trackers can be obtained, provided that they are excited, since oscillation occurs only about the local y axis, regardless of the oscillation amplitude, which mainly affects the predicted positions. The second step is still space scanning, however, with the specific orientation obtained in the first step. Although deviations in the excitation field direction and field strength still exist, as the field is optimized for the tracker orientation at the center of each subspace, it is sufficient to excite the tracker and yield a measured position close to the true value. After space scanning, a set of measured locations is obtained, clustered around the true position. The averaged value of these measurements, together with the orientation obtained in the first step, is then used as the initial guess for the third step. At this stage, both the orientation and approximate position of the trackers are available. In the third step, continuous excitation is performed with updated orientations and positions until a predefined maximum number of tracking iterations is reached, using the initial guess for the first iteration. Since the initial guess generally deviates from the true value, especially when the trackers are moving, multiple iterations may be required for the measurements to converge to a stable solution.

As validated in the previous section, accurate multi‐target localization can be achieved once the trackers are properly excited. Therefore, it is crucial to design an excitation platform that ensures proper excitation of the trackers. Here, a proof‐of‐concept excitation platform based on commercial coils is developed for multi‐target localization and tracking in 3D space. Three coils are arranged in a tri‐axial configuration with 120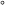 separation underneath the sensing array, as this represents the minimum requirement for generating an arbitrary 3D magnetic field. The coils can only be placed underneath the sensing array, as the region above the sensing array is reserved for patient access. The schematic of the setup is shown in Figure 4A. While there exists a magic angle of $∼54.74^{\circ }$ (the angle between the coil and the vertical axis) that can produce arbitrary magnetic fields at the focus center with equal efficiency in all directions [43], placing the coils at this angle would either position them too far from the focusing center or bring the focusing center undesirably close to the sensing array. Therefore, a 30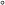 angle is adopted to achieve a focusing center that is sufficiently close to the coils while maintaining a distance from the sensing array. If the focusing center is too far from the coils, a much higher current is required to generate the target field. Conversely, if it is too close to the sensing array, the excitation height becomes limited, preventing excitation at larger distances. Figure 4A shows the coil arrangement with a 30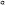 angle relative to the vertical axis. Assuming a 54.74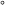 configuration is used, adjusting the coils would bring the focusing center closer to both the coils and the sensing array while keeping the coils underneath, whereas placing the coils at the edge of the array would increase both distances. With the 30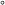 arrangement, the focusing center is located at distances of 140 and 80 mm from the coil center and the sensor plane, respectively. The required excitation field is generated as a linear combination of the fields produced by the coils, based on optimized current inputs derived from pre‐calculated field maps for this configuration. It should be noted that only positions along the coil axis are aligned with the field direction. Any positional deviation from the axis leads to a change in field orientation and must therefore be considered. Details of the field optimization are provided in the Experimental section. However, due to the relatively small coil size (outer diameter of 70 mm), the excitation field is most effective near the focusing center of the coil setup.

**FIGURE 4:**
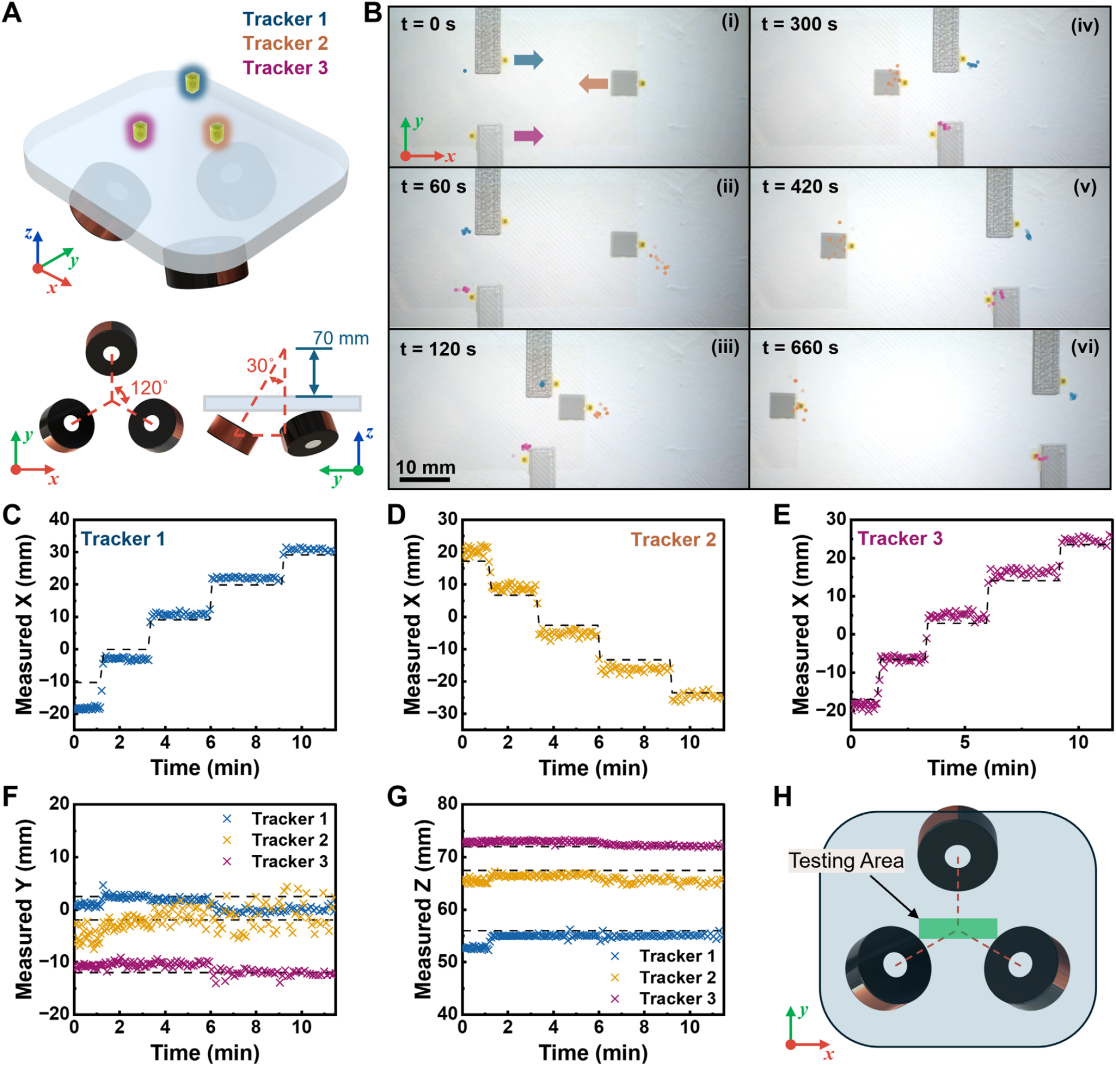
Continuous tracking with multiple trackers without initial information of tracker positions and orientations. (A) Schematic of the sensing and excitation platforms. (B) Snapshots at different time frames of three trackers moving along the global x axis. Images provide tracking results of the last 10 locations with color labels identical to (A). See also Video S2. (C–E) Measured X values of the three trackers, respectively. (F,G) Measured Y and Z values of the three trackers. (H) Approximated relative positions of the testing area to the sensing array and the excitation coils.

To demonstrate continuous tracking with multiple trackers, a three‐tracker experiment is conducted. Trackers 1 and 3 move in the same direction, while Tracker 2 moves in the opposite direction. They converge at the center and then separate, with Tracker 2 passing between Trackers 1 and 3, as shown in Figure 4B and Video S2. After each movement step, the trackers remain stationary for a short period before the next step. Figure 4B shows snapshots of the trackers at different time frames when they are stationary. The trackers move along the x axis, and therefore their Y and Z positions remain consistent. The real‐time tracking results are presented in Figure 4C–G. Figure 4C–E shows the measured X positions of the trackers, respectively. The measured values agree well with the ground truth (black dashed lines), except at the initial step of Tracker 1. The measured Y and Z positions are shown in Figure 4F,G, demonstrating good agreement with the ground truth during the period from 2 to 6 min, when the trackers are close to the system center. As the trackers move away from the center, tracking performance degrades due to limitations of the excitation platform. Consistent with previous observations, trackers with higher $f_{nat}$ exhibit better performance. Figure 4H illustrates the approximate travel region of the trackers. In these regions, the trackers are far from one coil and also deviate from the axes of the other two coils. Such positions lie outside the optimal operating region of the coil system and may require currents beyond practical limits, thereby pushing the field optimization away from the desired solution.

### 2.4 Miniaturized Wireless Trackers for Localized Rheological Measurements

In previous sections, we demonstrate multi‐target localization and continuous tracking using trackers that are quasi‐fixed by a strong adhesive. When applied to soft materials, particularly organ‐like tissues, the trackers may exhibit markedly different boundary conditions. Due to the intrinsic softness of these materials, both the resonance frequency and the damping rate are affected, with the damping rate increasing significantly compared to the quasi‐fixed condition. Moreover, the viscoelastic nature of these materials introduces strong coupling effects at high frequencies, further enhancing energy dissipation and leading to additional damping. These high‐damping conditions result in a rapid decay of oscillation, causing the measured signals to become very weak in the presence of any delay. Therefore, the signal amplitude must be compensated based on the damping rate extracted from the measured response. Nevertheless, these changes in the intrinsic dynamic properties suggest the potential of using such trackers as sensors for probing local variations in the mechanical properties of the surrounding environment [44].

To investigate the performance of the trackers in viscoelastic environments, gelatin gels with different concentrations are prepared, with trackers embedded in the gels to study how interactions with the surrounding material affect the tracker properties (Figure 5A). In this study, gelatin gels with concentrations ranging from 3% to 8% are used for performance evaluation, serving as simplified tissue‐mimicking materials with rheological properties comparable to biological tissues and commonly employed in organ phantoms [45, 46, 47]. In order to establish the relationship between the measured oscillation frequency, the damping rate and the underlying material properties, the storage and loss moduli ($G^{\prime }$ and $G^{\prime \prime }$) and complex viscosity ($\eta_{vis}$) of gelatin samples of different concentrations are characterized by measurements on a rotational rheometer. An approximately linear dependence of the modulus on gelatin concentration is observed on a logarithmic scale (Figure 5B). Consistent with this trend, the complex modulus and the complex viscosity also increase linearly in the logarithmic representation, as shown in Figure 5C. The measured properties are similar to real biological tissues, proving the feasibility of using such materials as tissue phantoms [48].

**FIGURE 5:**
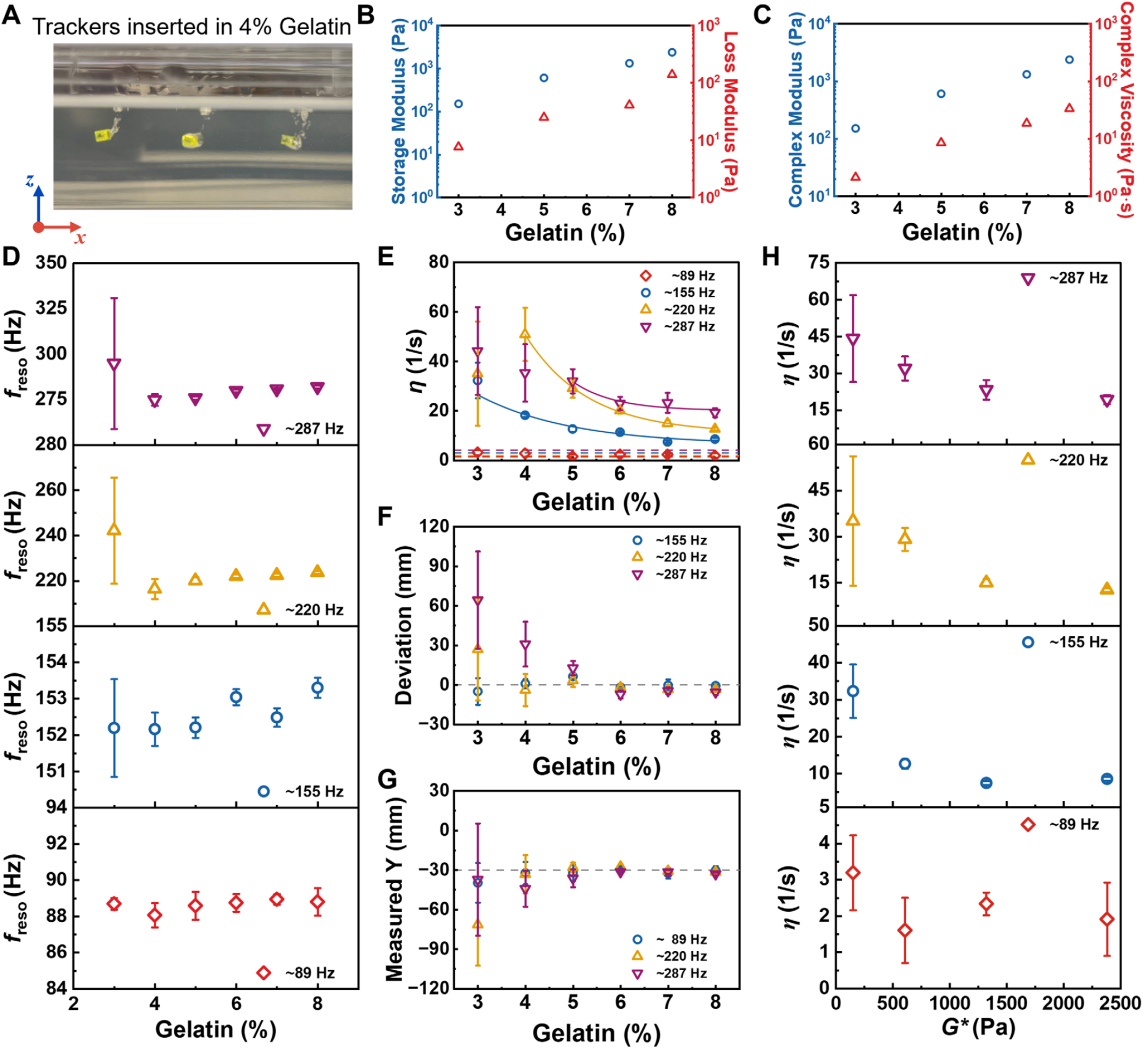
Miniaturized wireless trackers for localized rheological measurements. (A) Example of trackers inserted in gelatin gels for testing. (B) Storage and loss modulus of the gelatin gels at ∼10 Hz. (C) Complex modulus and complex viscosity of the gelatin gels at ∼10 Hz. (D) Resonate frequencies of the various trackers in gelatin gels with different concentrations. (E) Damping rates of the trackers, with various frequencies, in gelatin gels with different concentrations. (F) Deviations of the measured Z values from true values due to the soft gel environment. (G) Measured Y values of trackers deviate from the true value of −30 mm in soft gels. (H) Complex modulus mapping from the damping rate.

Since the mechanical properties of gelatin vary monotonically with concentration, a monotonic change in the tracker's oscillation frequency and damping rate would indicate the feasibility of establishing a mapping between the tracker response and the surrounding material properties. To verify this, the same three trackers as in the previous sections, with natural frequencies ($f_{nat}$) of approximately 155, 220, and 287 Hz, are used for localization tests after insertion into the different gelatin gels. An additional tracker with a natural frequency of approximately 89 Hz was also evaluated for comparison. As shown in Figure 5D, the relative variation in the operating frequency remains within approximately 5% over the measured range, except at very low gel concentrations where it is too soft for reliable measurements. Although the complex modulus influences both the resonance frequency and damping rate of the tracker, the tracker's response under the tested conditions is primarily dominated by damping‐related effects. The damping rate is thus used here as the primary parameter to establish the mapping to the complex modulus. Figure 5E shows the measured damping rates of the trackers, which increase as the gelatin concentration decreases for trackers with higher working frequencies. In contrast, the damping rate of the 89 Hz tracker remains relatively unchanged and close to the reference value, indicating weak coupling between the tracker and the surrounding medium. As a result, although the 89 Hz tracker maintains stable oscillatory behavior, its limited sensitivity makes it less suitable for localized rheological measurements. Moreover, trackers with higher natural frequencies exhibit higher damping rates under the same conditions. The localization results shown in Figure 5F,G further demonstrate that, under high damping conditions, the trackers fail to operate reliably, and the measured positions deviate from the ground truth with increased variability. As shown in Figure 5F, trackers with natural frequencies of approximately 155, 220, and 287 Hz can reliably measure gels of concentrations down to 4%, 5%, and 6%, respectively. In their functional regions, the measured damping rate shows large changes, indicating that these trackers can be used as miniature sensors for the localized rheological properties. The resulting mapping is presented in Figure 5H, where a monotonic relationship is observed, consistent with the monotonic variation of gelatin concentration. These results support the feasibility of using the damping response of the tracker for localized rheological sensing.

In general, this section demonstrates that trackers with a wide range of $f_{nat}$ will work for stiffer organ tissues, while a softer one only allows trackers with a low $f_{nat}$. That is, Tracker 1 works for both brain and liver tissues, while Tracker 3 only works for the liver tissue. However, since the properties of a tissue differ from region to region, it is preferable to have a tracker with a broader measurement range. A representative application scenario of the proposed tracker is the assessment of liver fibrosis, a pathological process known to induce pronounced changes in the mechanical environment of liver tissue [49, 50]. The associated stiffening, ranging from sub‐kilopascal values in healthy tissue to multiple‐fold increases in fibrotic liver, lies well within the range spanned by the gelatin models employed in this work [49, 51]. Note, although the tracker performance may degrade to some extent in very soft tissue environments, reliable localization in cancerous or tumorous regions remains feasible, as tumor‐associated tissues typically exhibit stiffness values that are several‐fold to an order of magnitude higher than those of normal soft tissues [52].

### 2.5 Multi‐Target Localization in ex vivo Porcine Liver

We further demonstrate multi‐target localization in porcine liver tissue using the proposed 3D search and tracking strategy without prior information on the trackers' position or pose. The trackers are inserted into the liver tissue using a 12G needle following a three‐step procedure: (i) needle insertion, (ii) tracker insertion, and (iii) tracker release followed by needle retraction, as illustrated in Figure 6A. Three trackers with natural frequencies of approximately 316, 220, and 287 Hz are employed. To enable ground‐truth comparison, the trackers are inserted from the inferior surface of the liver with controlled depths, avoiding the uncertainty associated with insertion from the convex superior surface. Figure 6B presents the localization results based on grouped cumulative averaging, where each hollow circle corresponds to the averaged position obtained from an increasing number of measurements from each cycle. The red, green, and blue cross symbols denote the true locations of Trackers 1, 2, and 3, respectively, assuming ideal vertical insertion. Figure 6C–E shows the accumulated average positions over time. Trackers 2 and 3 become relatively stable after the initial transient, although small systematic offsets from the ground truths (dashed lines) remain and vary across different coordinates. In contrast, Tracker 1 shows more pronounced fluctuations, particularly in the Z direction. This phenomenon is attributed to its higher natural frequency, which leads to a reduced signal‐to‐noise ratio under strong damping conditions in heterogeneous liver tissue. Figure 6F–H presents the localization errors between the accumulated average positions and the ground truth. The errors in X and Y gradually decrease and converge close to zero for all three trackers. The Z error also converges for Trackers 2 and 3, whereas Tracker 1 exhibits larger fluctuations and slower convergence. This is mainly due to the increased sensitivity of damping rate estimation to noise at low signal‐to‐noise ratios, which introduces variability in the parameter estimation and propagates to the position reconstruction. These results indicate that, under the current conditions, Trackers 2 and 3 are capable of continuous tracking, whereas Tracker 1 is more suitable for static localization due to its reduced stability. Nevertheless, the experiment successfully demonstrates multi‐target localization in porcine liver tissue. Overall, the fabricated miniaturized wireless trackers can function either as continuous trackers or as static locators, depending on the application requirements. The measured damping rates are 41.60±6.46 $s^{-1}$, 21.00±3.81 $s^{-1}$, and 29.23±2.27 $s^{-1}$ for Trackers 1 to 3, respectively. These values correspond to a material environment comparable to gelatin gels with concentrations of approximately 5%–6%, suggesting a complex modulus between 600–1000 Pa, consistent with reported values for porcine liver tissue (781–1117 Pa at 20–30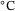) [48]. These results further indicate that the proposed trackers can serve as embedded miniature rheological probes, enabling in situ sensing of local mechanical properties through their dynamic oscillatory responses.

**FIGURE 6:**
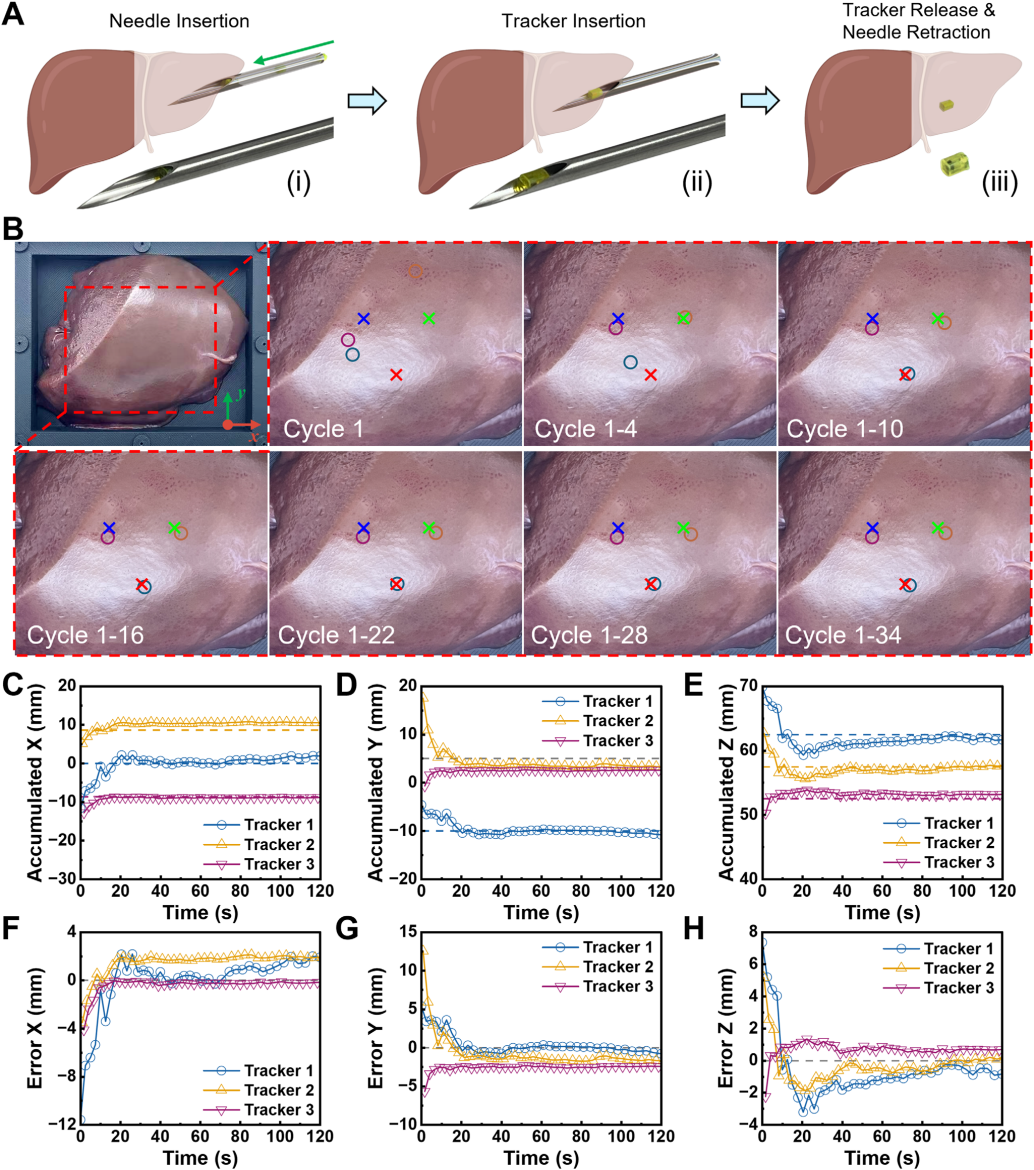
Multi‐target localization in ex vivo porcine liver. (A) Illustration of the deployment process of the trackers using a 12G needle. (B) Multi‐target localization results of three trackers embedded in the liver tissue, showing accumulated average positions over successive measurement cycles. Circles denote localization results, and crosses denote true positions. The colors red, green, and blue donate Trackers 1‐3, respectively. (C–E) Averaged X, Y, and Z positions of the trackers over time, respectively. (F–H) Corresponding localization errors in X, Y, and Z derived from (C–E), respectively.

### 2.6 Current Capabilities and Future Directions

The present study demonstrates the feasibility of miniaturized, frequency‐encoded SMOL trackers for wireless multi‐target localization and local mechanical sensing. Compared with our previous SMOL system, the reduced tracker size decreases the implantation footprint and facilitates the deployment of multiple trackers within a confined region. This miniaturization, however, also reduces the magnetic signal detected by the external sensor array, leading to an inherent trade‐off among tracker size, SNR, localization distance, accuracy, and temporal resolution. The practical lower size limit is therefore determined not only by the space required for cantilever oscillation, but also by the magnetic signal required for reliable localization. Similarly, the number of trackers is not fundamentally limited to three and can be increased when sufficiently separated resonance frequencies are available. With the current sequential‐excitation scheme, however, increasing the tracker number also increases the acquisition time and reduces the effective multi‐target refresh rate. In representative clinical tracking applications, three to five implanted fiducial markers have been employed, with the required number and spatial arrangement depending on the target geometry and tracking requirements [53, 54, 55]; accordingly, three trackers were used in the present study as a representative proof‐of‐concept demonstration of multi‐target SMOL. The present refresh rate of 0.6–1.3 Hz is therefore more suitable for continuous localization of quasi‐static or slowly varying targets than for high‐speed real‐time tracking. For local mechanical sensing, the present approach is particularly applicable to environments that provide sufficient mechanical constraint to the tracker, as demonstrated in the gelatin phantoms and *ex vivo* liver. Such measurements may potentially be useful for monitoring changes in local tissue mechanical properties associated with pathological processes such as liver fibrosis or cirrhosis.

The current system should be regarded as a proof‐of‐concept platform for demonstrating miniaturized multi‐target SMOL rather than an optimized application‐specific system. In particular, the three‐coil platform was developed to provide three‐dimensional excitation for the present validation. Because the required coil inputs depend on tracker position, excitation direction, operating frequency, and the constraints of the driving electronics, further extension of the excitation region will require system‐level optimization of the coil configuration, driving electronics, and control strategy. In addition, the weaker signals from the miniaturized trackers and the current sequential‐excitation strategy limit the temporal resolution. Future developments such as simultaneous multi‐frequency excitation, parallel signal processing, and more efficient or data‐driven localization algorithms may enable faster multi‐target localization.

In potential clinical applications, SMOL would serve a different role from established medical imaging modalities. Rather than replacing these techniques, it may complement them by enabling repeated localization of tracker‐associated targets without additional ionizing‐radiation exposure from the SMOL measurement itself. A further extension of the tracker could be its integration with untethered robotic systems, as preliminarily demonstrated in our previous SMOL work using millimeter‐scale robotic devices actuated by rotating and gradient magnetic fields [42]. Although robotic actuation is not investigated in the present study, combining the miniaturized trackers with independently optimized actuation mechanisms may allow their localization capability to support closed‐loop control of untethered robotic devices.

## 3 Conclusion

In summary, this work demonstrates the feasibility of miniaturized wireless trackers based on the small‐scale magneto‐oscillatory localization (SMOL) mechanism for multi‐target localization and local rheological measurement in biological soft tissues. By integrating tailored cantilever–magnet designs to achieve frequency‐selective oscillation (with natural frequencies ranging from ∼50 to ∼320 Hz), together with a proof‐of‐concept three‐coil excitation platform and a multi‐step excitation strategy, the system achieves sub‐millimeter precision and millimeter‐level accuracy over an operating distance up to 115 mm. Multi‐target tracking under quasi‐static conditions, along with successful localization in porcine liver tissue, validates the robustness of the approach in soft and deformable tissues. Furthermore, the frequency‐dependent dynamic responses of the trackers demonstrate their capability to function as miniaturized rheological probes for soft materials and biological tissues. Overall, these results highlight the potential of SMOL‐based wireless trackers as a radiation‐free solution for multi‐target localization, offering a compact and versatile platform for continuous spatial tracking and in situ rheological characterization in clinically relevant environments, particularly for minimally invasive procedures.

## 4 Experimental Section

### 4.1 Fabrication of Trackers

The trackers were fabricated with the following steps. First, the cantilevers with various thicknesses, including 5, 8, and 10 μm, were manufactured by an electrochemical etching process described in [56]. In brief, spring steel foils (Metall Ehrnsberger GmbH, Germany) with different thicknesses were etched in 200 mL of electrolyte prepared with 200 g of sodium chloride (Carl Roth, Germany) in 1 L of Milli‐Q water with a polymeric mask with shapes of the required cantilevers that was printed by a two‐photon polymerization (TPP) system (NanoOne 1000, UpNano GmbH, Austria). The etching process was achieved using a 2 V voltage (with respect to the double‐junction Ag/AgCl (Sigma–Aldrich, USA) reference electrode) to the working electrode with a frequency of 500 Hz and a duty cycle of 50%. The obtained cantilevers have a width of 200 μm and a length of 1.5 mm and are connected to a larger piece for the convenience of handling. Then, the cantilever was assembled with two micro‐magnets (NdFeB, N52, GMB Deutsche Magnetwerke GmbH, Germany) on both sides on an assisting stage to ensure 1 mm of free oscillating length of the cantilever before fixing the magnets to the cantilever with adhesive on the tip. The micro‐magnets are trapezoidal magnets with a top width of 200 μm, a bottom width of 300 μm, a height of 400 μm, and a thickness of 600 μm. Then, the resulted piece was fixed in between two polymeric pieces obtained by the NanoOne 1000 TPP machine with the UpPhoto resin (UpNano GmbH, Austria). This step is critical for assembly with housings, which avoids any direct touching of the fragile cantilever afterward. This obtained piece was inserted into two of the half‐housing structures, which were also printed with UpPhoto, and then sealed with adhesive to form the final tracker with an approximate volume of 4.6 ${\mathrm{mm}}^{3}$.

### 4.2 Finite Element Simulation of the Trackers

Finite element simulations were performed with comsol multiphysics (v6.1, COMSOL AB, Sweden) with different trackers to investigate the resonant frequency, excitation performance, interaction between multiple devices, and so on. All the simulations were performed with the Solid Mechanics module or coupled with magnetic fields. In general, the basic model has two or four attached micro‐magnets on a cantilever while the root of the cantilever is constrained with a spring foundation that lumps all boundary effects, such as a light housing weight, a soft surrounding environment, and a soft connection between the cantilever and the housing via adhesive, which cause deviations from an ideally fixed end. Oscillators with different types (2M and 4M) and different thicknesses were also simulated with different frequencies, strengths, and orientations of the excitation field to investigate the ideal responses of the oscillators. The interaction between multiple devices was studied by setting a fixed magnet part, sharing the same size as the oscillator's magnet, to the oscillator. Forces applied to the oscillator from an external magnet were calculated to obtain the safe distance between trackers without interactions changing the performance of the trackers.

### 4.3 Excitation and Sensing Platform

For tracker calibration and characterization, a single coil (LSIP‐330, Monacor International GmbH, Germany) was used to produce a uniaxial field with all trackers tested along its axis. For excitation in 3D space without initial information of the trackers' position and pose, three coils were used with a tri‐axial configuration with 120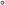 separation with all coil axes 30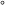 to the global z‐axis and converge at a point above the coil, located at x = y = 0. The sensing platform consists of a 20‐channel fluxgate sensor unit (FL1‐10‐20‐AUTO, Stefan Mayer Instruments, Germany) arranged in a 2D array with 10 sensors for x components and 10 for y components of the magnetic field. All the supporting frames for the excitation and sensing platforms were 3D‐printed with polylactic acid (PLA) using a 3D printer (Bambu Lab X1 Carbon, Bambu Lab, China). Details of the electrical setup of the system can be found in Supporting Information.

### 4.4 Tracker Calibrations

All fabricated trackers were calibrated with the protective housing fixed onto an ultra‐strong double‐sided adhesive to the testing stage to act as a quasi‐fixed environment, and only the internal quality of the trackers is assumed to affect the frequency of the resonant frequency and the damping rate. The calibration was performed by exciting the tracker at its resonant frequency with a maximum oscillating amplitude at a certain height above the sensing platform. The resonant frequency and the damping rate were calculated from the measured signal with 10 free oscillation periods after excitation.

### 4.5 Tracking with a Single Tracker with a Predefined Trajectory

The selected tracker and the excitation coil were aligned coaxially, and both were manually translated along a 3D‐printed PLA guide rail that defined letter‐shaped paths. Different letters of DKFZ were generated using trackers of different resonant frequencies to demonstrate frequency‐based trajectory encoding.

### 4.6 Three‐Dimensional Excitation

The three‐dimensional excitation was achieved by current optimization to obtain the required field for excitation at certain positions and orientations under maximum power limitations of the power amplifiers (A1110‐16‐E‐100, Dr. Hubert GmbH, Germany). The field generated by each coil was pre‐calculated based on a unit current input. Since magnetic fields are linear, the total magnetic field is the superposition of the fields generated by each coil.

### 4.7 Multi‐Target Tracking

Multi‐target tracking was implemented using a custom 3D‐printed stage equipped with a reverse helical transmission mechanism. Manual rotation of the stage drives the helical pathway, producing a synchronized opening‐closing motion of the displacement structure. Individual trackers were mounted on thin glass plates attached to the moving structure, thereby inducing well‐defined relative motions among the trackers. Continuous localization of all trackers was obtained by applying the multi‐step excitation scheme described earlier, enabling continuous tracking of multiple trackers under a quasi‐fixed environment.

### 4.8 Preparation and Rheology Measurements of Gelatin Gels

The rheological measurements were performed on gelatin from 3%∼8% wt/v, prepared with type A gelatin from porcine skin with a gel strength of ∼300 g Bloom (Sigma–Aldrich, USA) in Milli‐Q water, on a rotational rheometer (MCR 302, Anton Paar, Germany). The linear viscoelastic (LVE) range was determined, and the materials were tested by frequency sweep, ranging from 0.1 to 20 Hz, in oscillation tests.

### 4.9 Characterization in Gelatin Gels

Three resonant‐frequency‐differentiated trackers were embedded into individual gelatin gels at y≈−30 mm with spacing >10 mm to maintain near‐coaxial excitation. Their oscillatory responses were measured to determine the frequency and damping changes in the gel. Position reconstruction was performed by extrapolating the undamped signal amplitude from the measured damping because the increased damping in gelatin significantly reduced the effective signal amplitude within the usable excitation window after signal filtering.

### 4.10 Multi‐Target Localization in Porcine Liver Tissue

For multi‐target localization in tissue, freshly excised porcine liver obtained from a local butcher (Dürrröhrsdorfer Fleisch‐ und Wurstwaren GmbH, Germany) was placed in a custom 3D‐printed holder. The holder was designed with three pre‐defined insertion openings arranged in the xy‐plane at a radial distance of 10 mm from the center and spaced 120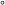 apart. Three trackers were inserted vertically into the liver through these openings using 12G needles, with their z‐axis insertion depths staggered by 5 mm. Three‐dimensional localization of all trackers was performed using the previously described multi‐step excitation scheme.

## Author Contributions

L.T., F.F., and T.Q. conceived the project. L.T. designed and developed the miniaturized wireless tracker system, performed the experiments, analyzed the data, and wrote the manuscript. J.K.M. performed rheological characterization and assisted with rheology‐related experiments and analysis. M.Z. and A.K. contributed to experimental design and results analysis. A.‐S.M. contributed to coding and computational implementation. F.F. developed the first‐generation SMOL framework and algorithm. T.Q. supervised the project and secured funding. All authors discussed the results and edited the manuscript.

## Conflicts of Interest

F.F. and T.Q. have a patent pending on the SMOL device (WO2024033464A1).

## Supporting information


**Supporting File 1**: advs78241‐sup‐0001‐SuppMat.pdf.

**Supporting File 2**: advs78241‐sup‐0002‐Video1.mp4.

**Supporting File 3**: advs78241‐sup‐0003‐Video2.mp4.

## Data Availability

The data that support the findings of this study are available from the corresponding author upon reasonable request.
