# Supplementary material for: Multi‐Target Wireless Localization of Miniaturized Magneto–Oscillatory Trackers in Soft Biological Tissues
Source: Adv Sci (Weinh). 2026 Nov 9:e78241. Online ahead of print. doi: 10.1002/advs.78241 (PMC13748781; doi:10.1002/advs.78241)
Supplement: Supplementary file 1 [file ADVS-9999-e78241-s001.pdf]

Supporting Information for Multi-target Wireless Localization of Miniaturized Magneto-oscillatory Trackers in Soft Biological Tissues

Liyuan Tan[1,2], Jyothi Kumari Mariyanna[1,3], Meng Zhang[1], Alexander Kutscher[1,2], Ann-Sophia Müller[1,4], Felix Fischer[1], Tian Qiu[1,2,3*]

[1]Division of Smart Technologies for Tumor Therapy,
German Cancer Research Center (DKFZ) Site Dresden, Dresden 01307, Germany.

[2]Institute of Semiconductors and Microsystems,
Faculty of Electrical and Computer Engineering, Dresden University of Technology (TUD), Dresden, Germany.

[3]Faculty of Medicine Carl Gustav Carus, Dresden University of Technology (TUD), Dresden, Germany.

[4]Faculty of Computer Science, Dresden University of Technology (TUD), Dresden, Germany.

*Corresponding author. Email: tian.qiu@dkfz.de

This PDF file includes:

Supplementary Text

Figures S1 to S7

Captions for Movies S1 to S2

Other Supplementary Materials for this manuscript:

Movies S1 to S2

Localization system

System control and data processing were performed in MATLAB (R2023b, The MathWorks, US) and Python (V3.12, Python Software Foundation, US) on an i9-13900K 3.00 GHz processor with 64 GB RAM and an NVIDIA GeForce RTX 4080 GPU. The control system was implemented using a PXI-based platform. The host computer communicated with the system through a PXIe-8398 remote control module connected to a PXIe-1092 chassis. Sensor signals were acquired via a PXIe-6349 data acquisition card with a sampling rate of 50 kS/s. The excitation coils were driven through a power amplifier controlled by the PXI system via a TB-4322 terminal block. The magnetic fields were measured with a 20-channel fluxgate sensor unit (FL1-10-20-AUTO, Stefan Mayer Instruments, Germany) with the individual sensors arranged in a 2D plane with a 3D-printed sensor positioning plate. For the excitation of the tracker, three electromagnetic coils (Monacor LSIP-330) were arranged beneath the sensor plate, oriented toward the center of the workspace with an angular separation of 120°. Each coil was tilted inward by 30° relative to the vertical direction, as illustrated in Figure 4A, to improve field focusing toward the central region. The coils were designed to generate a magnetic field above the coil (~1 mT at a distance of 30 mm) with full planar field-of-view. They were powered by three power amplifiers (Hubert A1110-16-QE-100, Germany) with a current amplitude of up to 10 A. Statistical analysis was performed in MATLAB. All experiments were performed at room temperature (~22 °C) in a shielded environment for external magnetic noise.

Simulations and analysis

Magnetic field generated by the excitation coils

The magnetic field generated by the excitation coils was characterized through a combination of numerical modeling and experimental measurements. The coil was modeled as a multi-layer, multi-turn circular winding, in which each turn was approximated as a current loop, and the total magnetic field was obtained by superposition based on the Biot–Savart law.

The number of effective turns was estimated from the measured electrical resistance of the coil, considering the resistivity of copper and a filling factor of 0.8. Based on this estimation, the coil was discretized into multiple radial layers and axial turns, and the magnetic field distribution was

calculated accordingly.

To improve the accuracy of the model, experimentally measured magnetic field data along the central axis were incorporated for calibration. As shown in Figure S7A and B, the measured and simulated magnetic field distributions in the xz-plane exhibit good agreement. The difference between the two is presented in Figure S7C, indicating that the overall field profile is well captured by the model with only minor local deviations. Furthermore, the comparison along the central axis (Figure S7D) shows strong consistency over a wide range of distances.

It should be noted that the magnetic field of a single coil was measured by translating a single fluxgate sensor along the axial direction, and only the axial component of the magnetic field was recorded. These measurements were used solely for model calibration. In contrast, during localization, a fluxgate sensor array was employed, as described in the main text.

Based on the validated model, the three-dimensional magnetic field distribution was further evaluated in the region above the coil. Orthogonal slices (Figure S7E–G) show a smooth and approximately symmetric field distribution, with a rapid decay along the axial direction and a relatively uniform field in the central region.

After establishing the calibrated magnetic field model for each individual coil, the field generated by each coil was precomputed and stored on a spatial mesh, including both magnitude and direction. During operation, the desired excitation field was synthesized by linearly combining the contributions from individual coils, and the corresponding coil currents were determined through an optimization process under the physical constraints of the system.

Finite element simulation of the oscillators

To obtain a better understanding of how the excitation magnetic field interacts with the trackers, and therefore provides feedback for improved selective excitation of multiple devices, finite element simulations are performed to investigate the oscillator motion upon the excitation of an external alternating magnetic field and the effect of an external permanent magnet that affects the performance of the oscillator when they are too close. The simulations are conducted using the commercial finite element analysis software Comsol Multiphysics with the coupling of the magnetic field and the solid mechanics.

For the excitation simulations, the geometrical parameters of the simulation model are identical

to the designed values for oscillator fabrication. A body-fixed magnetic moment is set with the oscillator during the oscillation, while a global external magnetic field is adopted to exert the torque. The applied magnetic field is given as follows.

$$B = B_0 \sin(2\pi f t) \tag{S1}$$

where B is the real-time field at time t, B_0 is the amplitude of the field, and f is the frequency of the field. Magnetic fields with various excitation frequencies when parallel to the cantilever, multiple field strengths, and at different angles to the oscillator are investigated. Based on the design, the maximum displacement of the centroid of the attached magnets along the perpendicular direction to the cantilever is 391.6 μm. However, due to fabrication errors and the unknown effective length of the cantilever, the targeting simulation values of the maximum displacement are between 300 to 350 μm. The appearance of the effective length of the cantilever arises from the actual center of rotation of the magnet. Before applying the time-varying field to the oscillators, eigenvalue studies are performed to find the resonant frequency of the oscillators, after which the frequency sweep can be conducted accordingly. All the simulations were obtained with a spring foundation rather than a fixed constraint to reproduce the non-ideally fixed oscillators during testing and real application scenarios. By comparing the simulation results and the experimental results of oscillators with different cantilever thicknesses, a spring constant of 1×10^6 N/m is approximated as a baseline for general cases.

Excitation with non-perpendicular magnetic fields

Ideally, the excitation field is applied perpendicular to the magnetic moment of the oscillator to achieve the maximum oscillation amplitude and thus the strongest detection signal. However, in practice, it is not necessary to precisely align the excitation field with the optimal direction, as long as the induced oscillation is sufficient for reliable localization.

To investigate the effect of misalignment, simulations were performed for different yaw (φ) and pitch (θ) angles of the excitation field, using an oscillator with a cantilever thickness of 8 μm. Due to symmetry, only one octant of the angular space was simulated. Figure S4B shows the heatmap of the maximum oscillation amplitude within this octant, and Figure S4C presents the corresponding distribution mapped onto a unit sphere.

As shown in Figure S4B, the maximum achievable amplitude decreases with increasing pitch angle θ, while the yaw angle φ has a relatively minor effect. Figure S4D further compares simulation and experimental results as θ increases at $\varphi = 0°$. The amplitude decreases monotonically with θ, and no oscillation is observed at $\theta = 90°$. This is because the excitation field becomes parallel to the magnetic moment, resulting in negligible effective torque, or induces torsional motion rather than bending.

For $\theta < 90°$, the excitation field retains a component along the effective bending direction of the cantilever (defined as the z-axis), which drives the oscillation. The fitted curve in Figure S4D follows a cosine function, consistent with the projection of the excitation field along this axis. Therefore, the normalized oscillation amplitude can be expressed as

$$\frac{A}{A_0} = \cos(\theta), \tag{S2}$$

where A is the maximum oscillation amplitude at pitch angle θ, and A_0 is the amplitude at $\theta = 0°$.

The simulation results agree well with the experimental measurements, which are normalized by the simulated amplitude at $\theta = 0°$. This relationship indicates that moderate misalignment still allows efficient excitation. For example, at $\theta = 30°$, the normalized amplitude remains $A/A_0 = \cos(30°) \approx 0.866$, corresponding to 86.6% of the maximum amplitude.

To investigate how the trackers will perform with an excitation field that is not completely perpendicular to the magnetic moment and instead has a misalignment angle to the tracker's axis, the trackers (as used in Figure 3) are tested with different angles about their local y axis while still positioned on the axis of the excitation coil, as shown in Figure S3E. The results presented in Figure S3F to Figure S3H show a slightly larger deviation from the ground truth in measured X and Y positions, while it is much more pronounced in measured Z. This is attributed to the reduced component of the excitation field perpendicular to the tracker's magnetic moment.

Consequently, the field cannot drive the tracker to its maximum amplitude (limited by the housing), resulting in a reduced signal and an overestimation of the Z position. This effect can be mitigated by increasing the excitation cycles or the overall field strength to ensure sufficient excitation.

Finite element simulations and experiments are conducted to study how excitation misalignment and field strength affect the maximum amplitude under 10 excitation cycles. As can be seen

in Figure S4 and Figure S5, the amplitude follows the cosine relationship with respect to the angle of misalignment, and it increases linearly with the applied field. Therefore, a misalignment angle of 15–30° is acceptable if the excitation cycles and field strength are set above the minimum required to achieve the maximum amplitude.

Fibonacci spherical sampling

The Fibonacci spherical sampling is adopted to avoid excessive sampling at small pitch angles. The sampling method uses points uniformly distributed on a spherical surface that reduces the scanning time and therefore results in a faster refresh rate. Considering a μSMOL device with unknown orientation, at least a semi-sphere region has to be scanned due to symmetry. Then, the discretization of the semi-sphere can be obtained as follows.

First, the number of discretization points is calculated. Since we already know that a deviation angle of 30° still preserves a strong oscillation amplitude, this angle can be used as the control angle for discretization. Assuming the semi-spherical surface is discretized with N points, the average area A_{pt} that a point occupies can be calculated as

$$A_{pt} = \frac{2\pi}{N}. \tag{S3}$$

The equivalent spherical crown area with an angle of θ that occupies an identical area can be expressed as

$$A_{cr} = 2\pi(1 - \cos(\theta)). \tag{S4}$$

Thus, the approximated number of discretization points can be obtained by equating Equation S3 and Equation S5 and therefore the number of points can be expressed as

$$N = \frac{1}{1 - \cos(\theta)}. \tag{S5}$$

Since the number of points must be an integer, the value is rounded up to the nearest integer. For example, if $\theta = 30°$ is used, then the number is $N = 7.464 \approx 8$. Therefore, the number of points used for generating the Fibonacci sampling points of a semi-spherical surface is 8. For each point from $i = 0, 1, 2, ..., N - 1$, the z value from the north pole to the equator is

$$z_i = 1 - \frac{i}{N-1}, \tag{S6}$$

with a corresponding radius r_i in the xy plane of

$$r_i = \sqrt{1 - z_i^2}, \tag{S7}$$

and a corresponding yaw angle φ_i of

$$\varphi_i = \phi \cdot i, \tag{S8}$$

where $\phi = \pi(3 - \sqrt{5}) \approx 2.34$ rad is the Golden Angle. Then the corresponding x_i and y_i coordinates can be obtained by

$$x_i = r_i \cos(\theta_i), \tag{S9}$$

and

$$y_i = r_i \sin(\theta_i). \tag{S10}$$

Figure S6A shows the number of sampling points and the change of rate from a sweep of pitch angles (θ). It is expected that the larger the angle, the smaller the sampling number. From the rate plot, one can find that the decreasing rate becomes slower when θ is higher than 20°. The number of sampling points has significantly dropped from 263 at $\theta = 5°$ to 17 at $\theta = 20°$. Using the boundary value of $\theta = 30°$ further reduces the number to 7. However, an overlap region should be considered to avoid practical singularity. Considering an overlap angle of 5° ($\theta = 25°$) or 10° ($\theta = 20°$) gives a sampling number of 11 or 17, respectively, without significantly increasing the sampling number when compared with 8 points at $\theta = 30°$. The orientations and distributions of the sampling points for each case are provided in Figure S6B and Figure S6C.

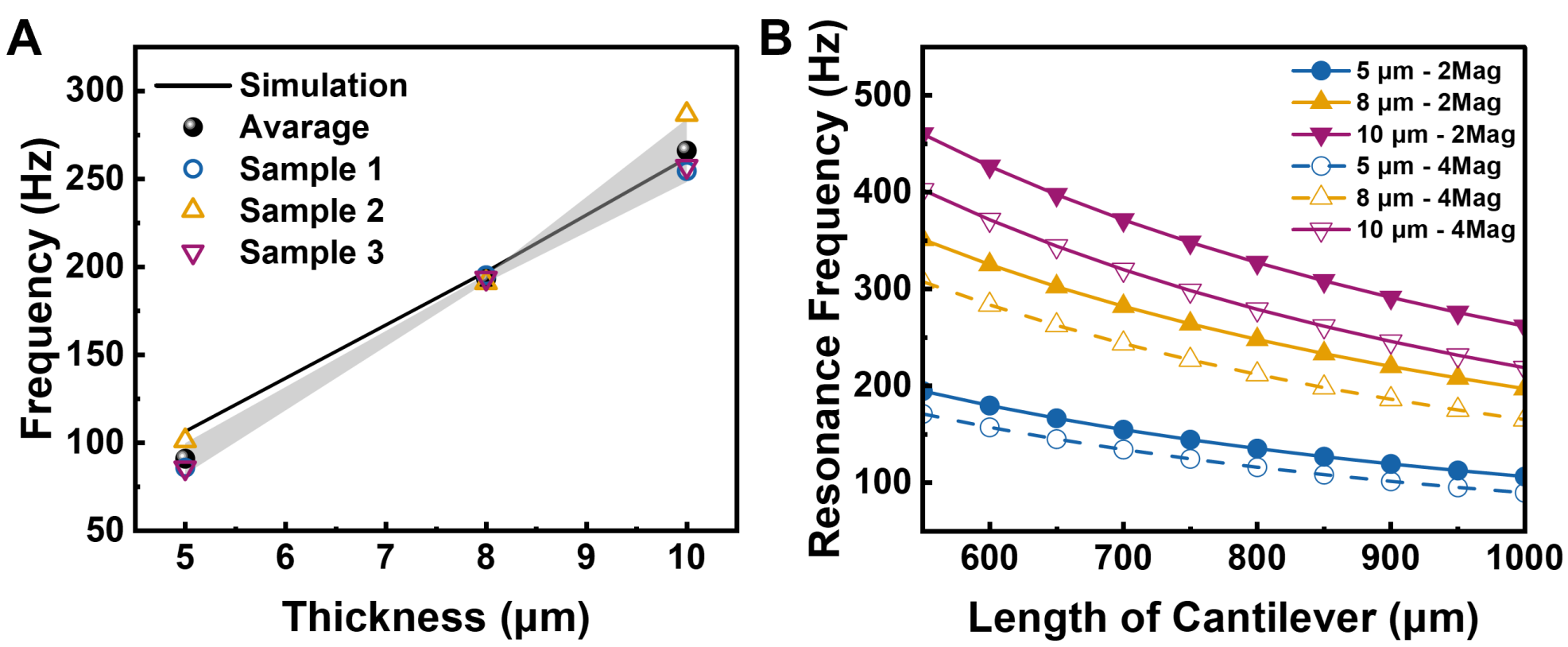

Figure S1: **Resonance frequency dependence on structural parameters and magnetic configuration.** (A) Comparison between simulated and experimentally measured resonance frequencies for oscillators with different thicknesses under the 2M configuration. The measured results (individual samples and average) agree well with the simulation. (B) Simulated resonance frequencies as a function of cantilever length for different thicknesses under both 2M and 4M configurations. The resonance frequency decreases with increasing cantilever length due to reduced structural stiffness. In addition, the 4M configuration exhibits lower resonance frequencies than the 2M case because the added magnetic elements increase the effective mass of the oscillator.

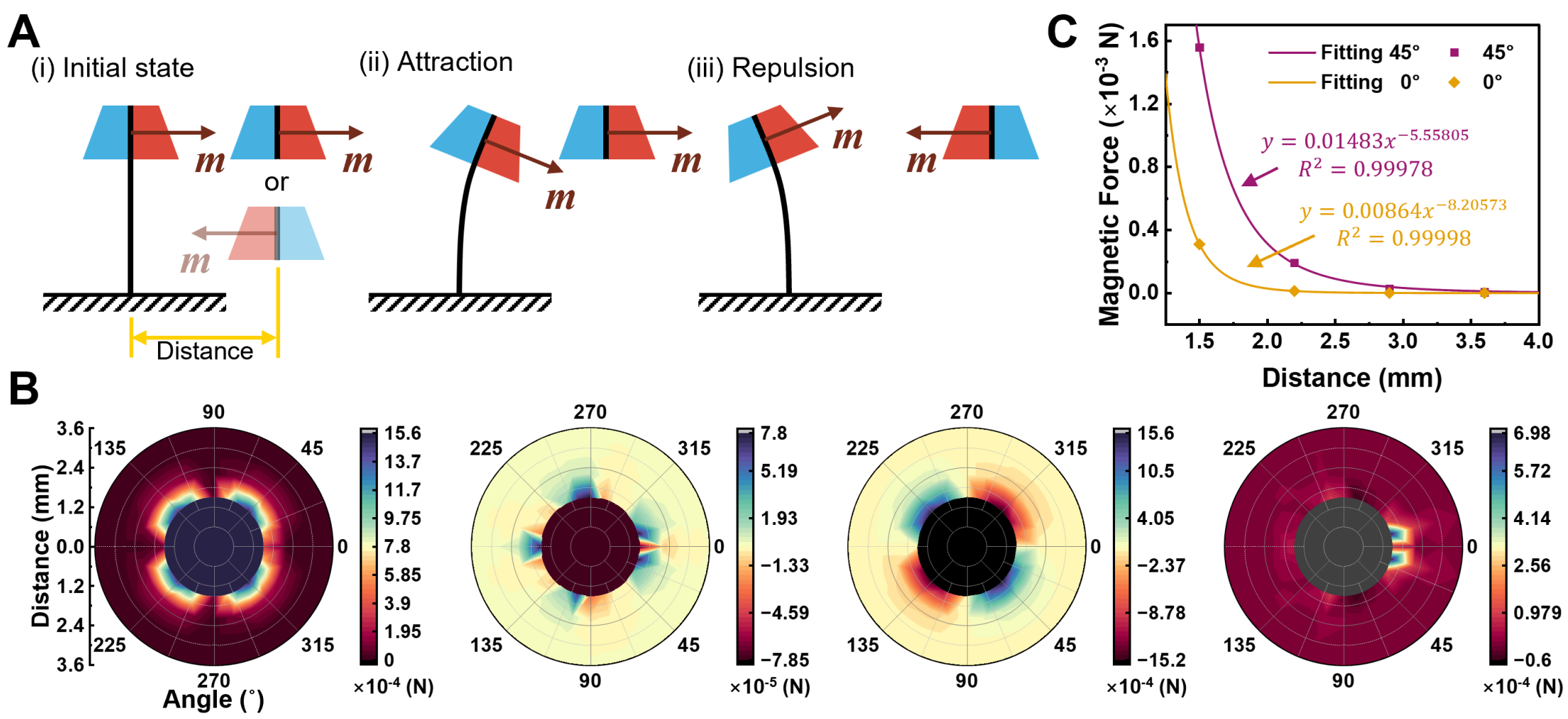

Figure S2: **Finite element simulation of the working distance based on magnetic interactions between devices (2M case).** (A) Schematic illustration of the interaction between two magnetic elements, showing the initial state, attraction, and repulsion configurations depending on their relative orientation. (B) Simulated angular distribution of the magnetic force at different distances, presented in polar coordinates. The full-field distributions are reconstructed using symmetry. (C) Total magnetic force as a function of distance for aligned (0°) and tilted (45°) configurations, with power-law fitting. The rapid decay of the interaction force indicates that a minimum separation of approximately 4 mm is required for the 2M case to avoid strong magnetic coupling. For multi-device configurations, this constraint scales accordingly, requiring at least ~8 mm for a 2M+4M case and ~16 mm for a 4M+4M case. A conservative margin of an additional ~2 mm is further considered in practice.

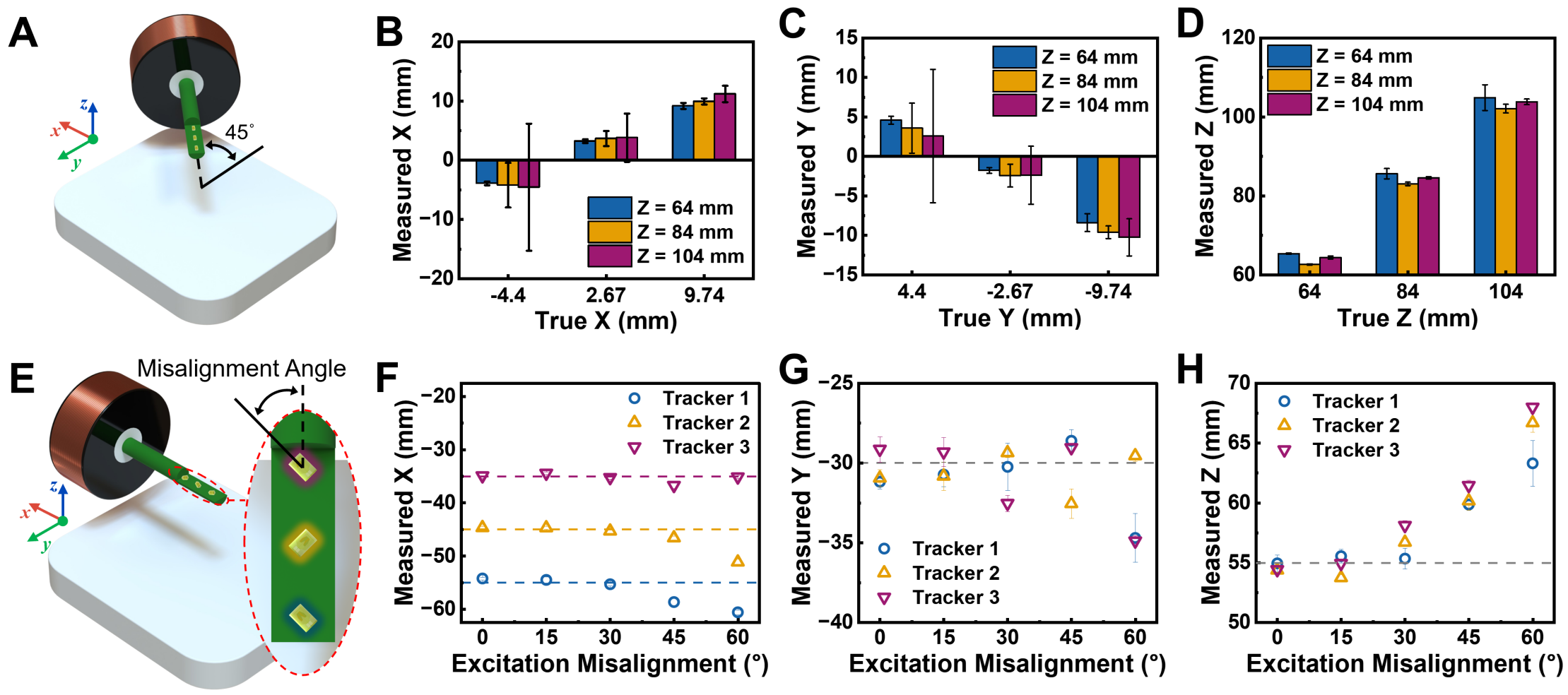

Figure S3: **Localization performance under a 45° tilted configuration and with excitation misalignment.** (A) Schematic illustration of the tracker tilted at 45° toward the center. The same device as used in Figure 3 is adopted. (B–D) Comparison between measured and true positions along the x, y, and z directions at different heights (z = 64, 84, and 104 mm). Overall, the measured positions agree well with the ground truth. However, the localization accuracy decreases with increasing distance, with more pronounced errors observed in the lateral directions (x and y), particularly for the lower-frequency tracker. (E) to (H) Schematic and localization results with trackers excited with a misaligned angle to the axis of the excitation coil with true positions identical to Figure 3E. All results are presented with N = 20.

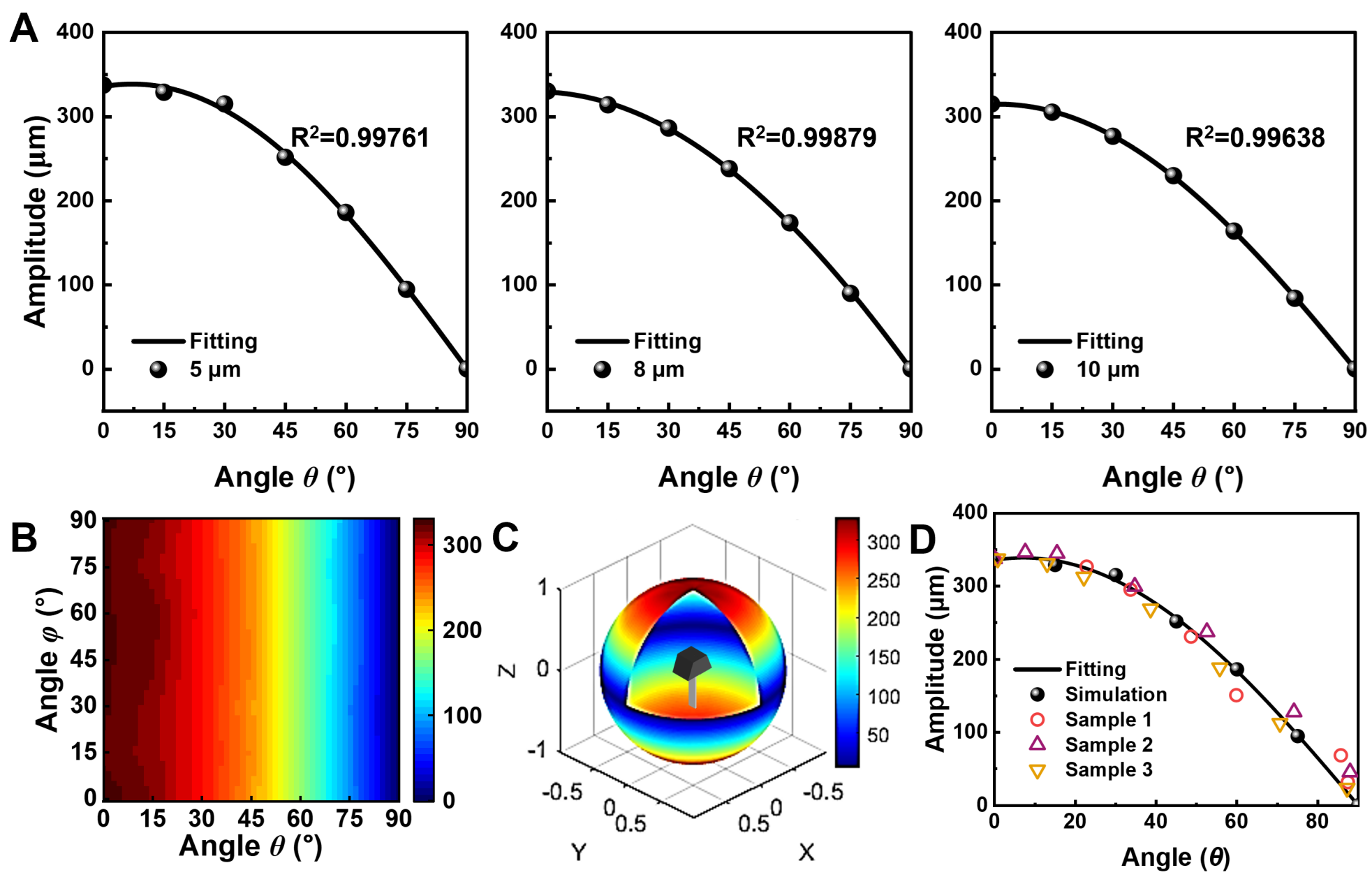

Figure S4: **Angular dependence of the bending amplitude under misalignment between the magnetic field and the magnetic moment.** (A) Simulated bending amplitude after 10 oscillation cycles as a function of the misalignment angle θ for oscillators with different thicknesses (5 μm, 8 μm, and 10 μm). The results follow a cosine dependence and are well fitted by a cosine function. (B) Two-dimensional map of the bending amplitude as a function of the misalignment angle, illustrating the continuous reduction in excitation efficiency with increasing θ. (C) Three-dimensional visualization of the angular dependence, highlighting the projection effect of the magnetic torque. (D) Comparison between simulation and experimental measurements for three samples after amplitude normalization, showing that despite differences in absolute amplitude due to device variations, all datasets collapse onto a consistent cosine trend. Note that the torsional deformation of the cantilever induced by large misalignment angles, which may lead to oscillation instability, is not considered in this analysis.

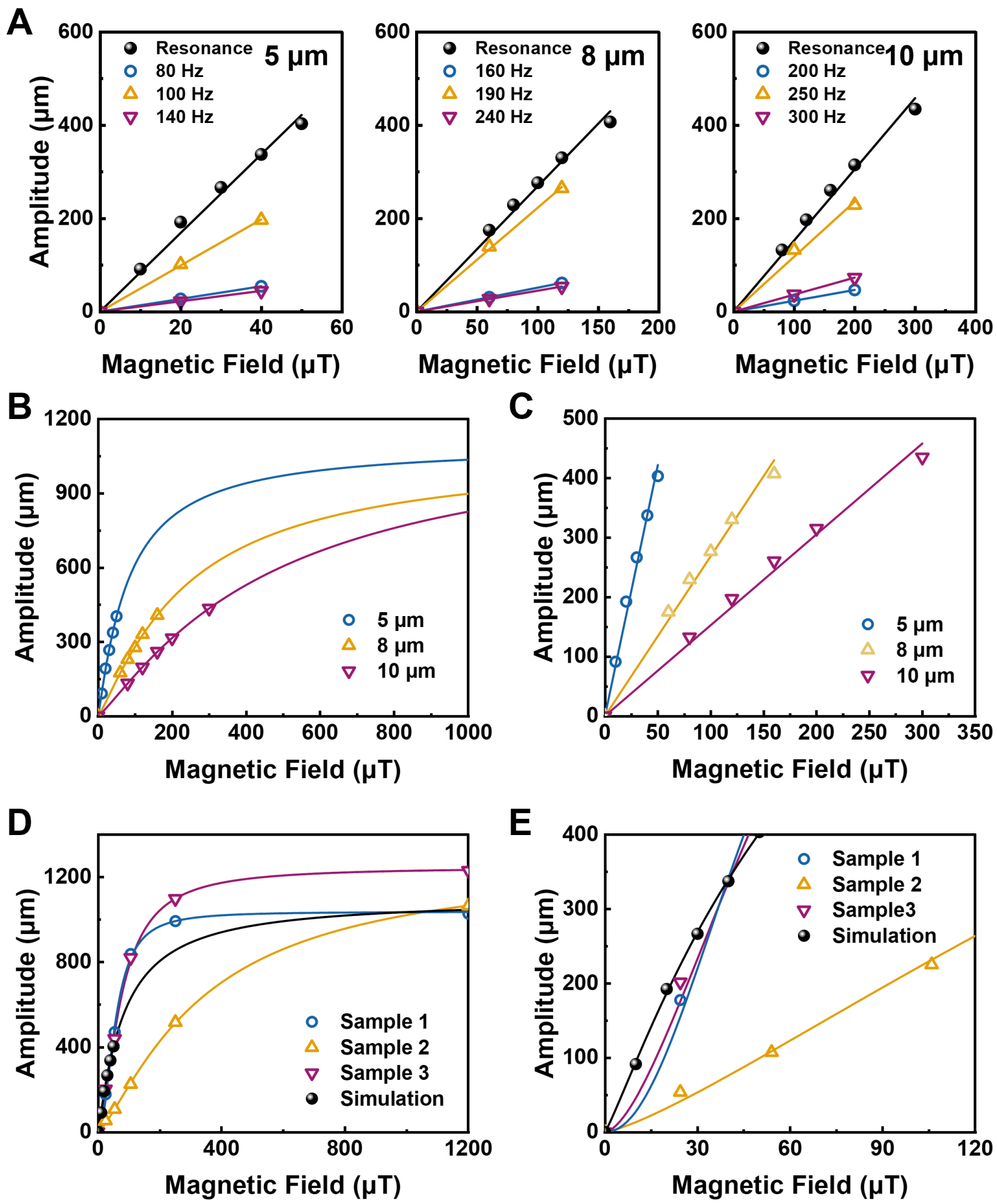

Figure S5: **Magnetic-field-dependent bending amplitude of the oscillator under excitation.** (A) Simulated bending amplitude after 10 oscillation cycles as a function of magnetic field strength for oscillators with different thicknesses (5 μm, 8 μm, and 10 μm). Results at multiple excitation frequencies around resonance are shown, highlighting the frequency-dependent dynamic response. (B) Simulated bending amplitude under resonant excitation over an extended magnetic field range, showing a nonlinear response that gradually approaches saturation. (C) Enlarged view of (B) in the low-field regime with linear fitting, demonstrating a linear relationship between magnetic field strength and bending amplitude at small displacements. (D) Comparison between simulated results and experimental measurements for three samples, showing good agreement in the nonlinear regime. (E) Comparison between simulation and experimental results in the low-field regime, confirming the linear response behavior across different samples.

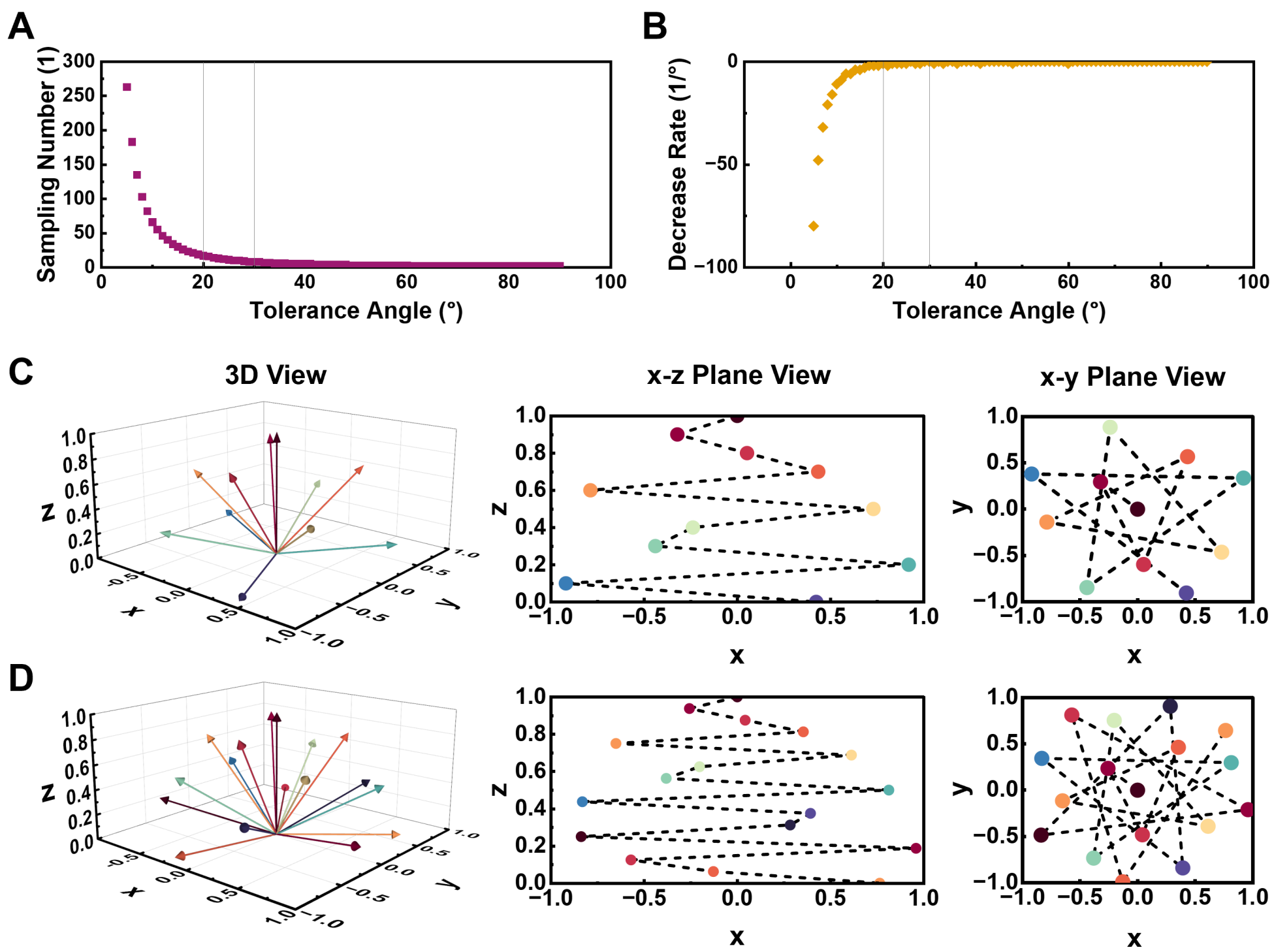

Figure S6: **Fibonacci spherical sampling and its dependence on tolerance angle.** (A) Number of required sampling directions as a function of the tolerance angle. (B) Corresponding decrease rate of the sampling number with increasing tolerance angle. (C) Visualization of the sampling directions generated by the Fibonacci sphere method, shown in 3D view and their projections onto the xz- and xy-planes. (D) Example of a denser sampling configuration under a smaller tolerance angle, presented in 3D view and corresponding planar projections, illustrating improved angular coverage.

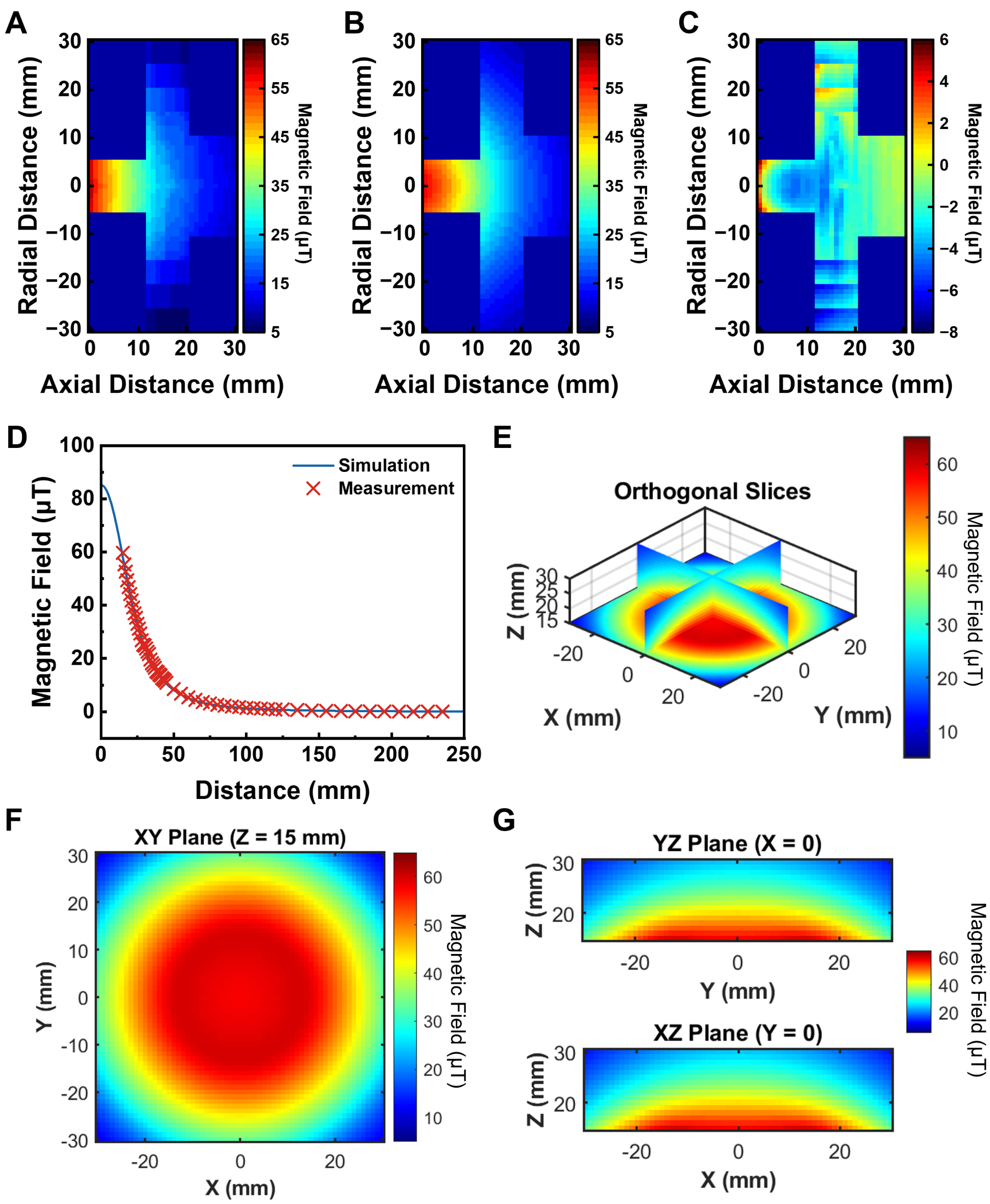

Figure S7: **Experimental measurement and simulation of the magnetic field generated by the LSIP-330 coils.** (A) Experimentally measured magnetic field distribution in the xz-plane under an applied current of 0.01 A. (B) Simulated magnetic field distribution in the same xz-plane, showing good agreement with the experimental results in (A). (C) Difference between the measured and simulated magnetic fields, highlighting the overall consistency and local deviations. (D) Magnetic field magnitude along the central axis as a function of distance, comparing simulation (blue line) and experimental measurements (red crosses). (E) Three-dimensional visualization of the simulated magnetic field distribution using orthogonal slices. (F) Simulated magnetic field distribution in the xy-plane at $z = 15$ mm. (G) Simulated magnetic field distributions in the yz-plane ($x = 0$) and xz-plane ($y = 0$), respectively.

Caption for Movie S1. Tracking trajectories of individual miniaturized trackers with different natural frequencies. The video shows the tracking results of four individual trackers with different natural frequencies (57 Hz, 192 Hz, 287 Hz, and 316 Hz) during magnetic localization experiments. Colored dots represent the reconstructed positions of the tracker over time, with the color map indicating temporal progression from t_0 to t_n. The corresponding trajectory of each tracker by optical tracking is overlaid to generate Figure 2G.

Caption for Movie S2. Continuous tracking of multiple miniaturized trackers. The video demonstrates the continuous localization and tracking of multiple trackers operating at different resonance frequencies within the same workspace. Different colors correspond to different trackers. For each tracker, the displayed trajectory includes the current reconstructed position together with several previous positions, where temporal progression is represented by transparency, with older positions shown more transparently. The experiment highlights the capability of frequency-selective magnetic localization for the continuous tracking of multiple miniaturized trackers. Snapshots of this video are shown in Figure 4B.
